# Transnational inequities in cardiovascular diseases from 1990 to 2019: exploration based on the global burden of disease study 2019

**DOI:** 10.3389/fpubh.2024.1322574

**Published:** 2024-04-03

**Authors:** Ben Hu, Jun Feng, Yuhui Wang, Linlin Hou, Yinguang Fan

**Affiliations:** ^1^Department of Cardiology, The Second People's Hospital of Hefei, Hefei Hospital Affiliated to Anhui Medical University, Hefei, Anhui, China; ^2^The Fifth Clinical Medical School of Anhui Medical University, Hefei, Anhui, China; ^3^Department of Epidemiology and Biostatistics, School of Public Health, Anhui Medical University, Hefei, Anhui, China

**Keywords:** disability-adjusted life-years (DALYs), health inequality, cardiovascular disease, socio-demographic index, slope index of inequality, concentration index

## Abstract

**Background:**

To describe the burden and examine transnational inequities in overall cardiovascular disease (CVD) and ten specific CVDs across different levels of societal development.

**Methods:**

Estimates of disability-adjusted life-years (DALYs) for each disease and their 95% uncertainty intervals (UI) were extracted from the Global Burden of Diseases (GBD). Inequalities in the distribution of CVD burdens were quantified using two standard metrics recommended absolute and relative inequalities by the World Health Organization (WHO), including the Slope Index of Inequality (SII) and the relative concentration Index.

**Results:**

Between 1990 and 2019, for overall CVD, the Slope Index of Inequality changed from 3760.40 (95% CI: 3758.26 to 3756.53) in 1990 to 3400.38 (95% CI: 3398.64 to 3402.13) in 2019. For ischemic heart disease, it shifted from 2833.18 (95% CI: 2831.67 to 2834.69) in 1990 to 1560.28 (95% CI: 1559.07 to 1561.48) in 2019. Regarding hypertensive heart disease, the figures changed from-82.07 (95% CI: −82.56 to-81.59) in 1990 to 108.99 (95% CI: 108.57 to 109.40) in 2019. Regarding cardiomyopathy and myocarditis, the data evolved from 273.05 (95% CI: 272.62 to 273.47) in 1990 to 250.76 (95% CI: 250.42 to 251.09) in 2019. Concerning aortic aneurysm, the index transitioned from 104.91 (95% CI: 104.65 to 105.17) in 1990 to 91.14 (95% CI: 90.94 to 91.35) in 2019. Pertaining to endocarditis, the figures shifted from-4.50 (95% CI: −4.64 to-4.36) in 1990 to 16.00 (95% CI: 15.88 to 16.12) in 2019. As for rheumatic heart disease, the data transitioned from-345.95 (95% CI: −346.47 to-345.42) in 1990 to-204.34 (95% CI: −204.67 to-204.01) in 2019. Moreover, the relative concentration Index for overall CVD and each specific type also varied from 1990 to 2019.

**Conclusion:**

There’s significant heterogeneity in transnational health inequality for ten specific CVDs. Countries with higher levels of societal development may bear a relatively higher CVD burden except for rheumatic heart disease, with the extent of inequality changing over time.

## 1 Introduction

Cardiovascular diseases (CVD) are among the most prevalent chronic diseases, comprising ten major subtypes. They are leading causes of mortality and morbidity globally, imposing significant economic burdens on patients, their families, healthcare services, and societies (1, 2). High disease burden is often attributed to underdevelopment, limited access to healthcare systems, and suboptimal healthcare performance. For CVD, transitions in lifestyle factors like diet, physical activity, and smoking, coupled with environmental pollution, lay the foundation for CVD onset (3–6). Emerging trends indicate that many developed countries face epidemics of obesity and diabetes, which exacerbate most CVD risk factors and negatively impact cardiac structure and function (7, 8). Industrial and technological revolutions and associated economic and societal changes have shifted disease burdens from infectious diseases and malnutrition in the 20th century to CVD in most high-income nations (9). The progression of these industrial developments, exposure to risk factors, and aging underscore that CVD remains a significant public health concern (10, 11). Assessing and monitoring health inequalities of CVD are crucial for achieving health equity, a core objective of the 2030 Sustainable Development Agenda (12). Disparities in health across nations with varying levels of societal development warrant exploration.

In epidemiology, health inequality monitoring utilizes health-related data to inform policies and initiatives aimed at addressing health disparities. Simultaneously, it establishes a numerical quantification of inequality within populations based on categorized data, facilitating comparisons of health disparities across different time periods, backgrounds, and indicators. Previous research has focused on measuring overall inequality using standards such as the Gini coefficient, which only consider the distribution of health indicators within the population (13). However, social inequality metrics need to assess how health indicators vary based on different demographic, socioeconomic, or geographical characteristics. In addition, in terms of monitoring health inequality, the term “brief measures of health inequality” generally refers to measures of social inequality, as this is the primary focus of attention. There are numerous summary measures, each with distinct characteristics, which can lead to different conclusions about the degree and direction of inequality (14, 15).

In order to better account for the impacts of population, economy, and country, we used the World Health Organization (WHO) recommended standard methods for health equity analysis including two indicators of absolute and relative inequality, it further delves into the analysis of transnational inequalities related to cardiovascular diseases associated with Socio-Demographic Index (SDI) (16). This approach is taken to investigate whether disparities related to societal development levels exist in the burden of these CVDs across countries, determining their severity and how they have evolved over time.

## 2 Methods

### 2.1 Data source

The methodological details of the 2019 Global Burden of Diseases, Injuries, and Risk Factors Study (GBD) have been previously published (1, 17, 18). These studies detailed the burdens of 369 diseases and injuries in 204 countries and regions, broken down by gender and age group from 1990 to 2019, as well as 87 risk factors divided by incidence, prevalence, deaths, and disability-adjusted life-years (DALYs). We procured estimates of DALYs, specifically for CVD and ten specific CVDs, including rheumatic heart disease, ischemic heart disease, stroke, hypertensive heart disease, non-rheumatic valvular heart disease, cardiomyopathy and myocarditis, atrial fibrillation and flutter, aortic aneurysm, peripheral artery disease and endocarditis (19), for the years 1990 to 2019, and their 95% uncertainty intervals (UI) for analysis from the Global Health Data Exchange (GHDx) query tool.^1^ The definition of specific CVD used the codes from the tenth edition of the International Classification of Diseases (ICD-10). The definition of disease is shown in Supplementary Table S1. In addition, the socio-demographic index (SDI) is a comprehensive indicator that measures a country’s or region’s level of development based on factors such as fertility rate, education level, and *per capita* income, with an SDI ranging from 0 to 1. The SDI is reportedly associated with disease incidences and mortality rates. It quantifies the level of social and population development in a country or region (20, 21). Detailed data for the population and economic status of all CVDs in all countries and territories are available in Supplementary Table S2. Given the reliance on public datasets, this study was exempted from the Ethics Committee of the Second People’s Hospital of Hefei.

### 2.2 Burden description

DALYs are conceptualized as healthy life years lost due to a specific disease, representing the difference between an individual’s current and optimal health status—the latter being a life expectancy free from disease or disability (22). Descriptive analysis was deployed to assess the global burden and its health implications for overall and specific CVD. We computed the cases of DALYs for 1990 and 2019, the rate of DALYs per 100,000 population, and the shift in this burden. The burden of overall and specific CVD across various global regions was compared for 1990 and 2019.

### 2.3 Cross-country inequality analysis

Two distinct metrics of absolute and relative inequalities were utilized to gauge cross-country inequality in the CVD burden: the Slope index of inequality (SII) and the relative concentration index (16, 23, 24). Absolute inequality reflects the magnitude of the difference in health between countries with different SDIs (23). The SII represents the absolute difference in predicted values of burden between those with the highest level of SDI and those with the lowest level of SDI, while taking into consideration the entire distribution of SDI using an appropriate regression model. It was computed by regressing national-level burdens owing to CVD in all age population on a scale of relative social position, defined by the midpoint of the cumulative class interval of the population ranked by SDI. Because of heteroskedasticity, robust regression was used: repeated iterative weighting. SII assumes the value of zero. Greater absolute values indicate higher levels of inequality. Positive values indicate a concentration of the indicator among the advantaged and negative values indicate a concentration of the indicator among the disadvantaged (16). Therefore, when the SII is positive, the burden is concentrated in countries with higher SDI; When the SII is negative, the burden is concentrated in countries with lower SDI. Relative inequality measures show proportional differences in health among countries with different SDIs (25). The concentration index is a relative measure of inequality that shows the health gradient across multiple subgroups with natural ordering (according to SDI). It indicates the extent to which burden is concentrated among the disadvantaged (population with low SDI) or the advantaged (population with high SDI). It was derived through numerical integration beneath the Lorenz concentration curve, which was fitted using the DALYs cumulative percentage against the relative cumulative distribution of the population sorted by SDI (25). Given that a population is ranked by increasing socioeconomic status. When a Lorenz curve above the line of equality, it indicates health burdens concentrated in lower-income countries, represented by a negative value of the concentration index. When there is no inequality, the concentration index is 0 (24).

### 2.4 Statistical analysis

All analyses and visualizations were performed using the Health Equity Assessment Toolkit from WHO (16) and the R software (V.4.3.0). The R software V.4.3.0 and the WHO’s Health Equity Assessment Toolkit were used to calculate the SII and the relative concentration index and to estimate their 95% CIs. Robust regressions were conducted using the “MASS” package in R V.4.3.0, with the “car” package checking for heteroscedasticity in the fitted models, and the “ggplot2” package was utilized for disease plotting and data visualization.

## 3 Results

### 3.1 Cardiovascular diseases

**Figure 1:**
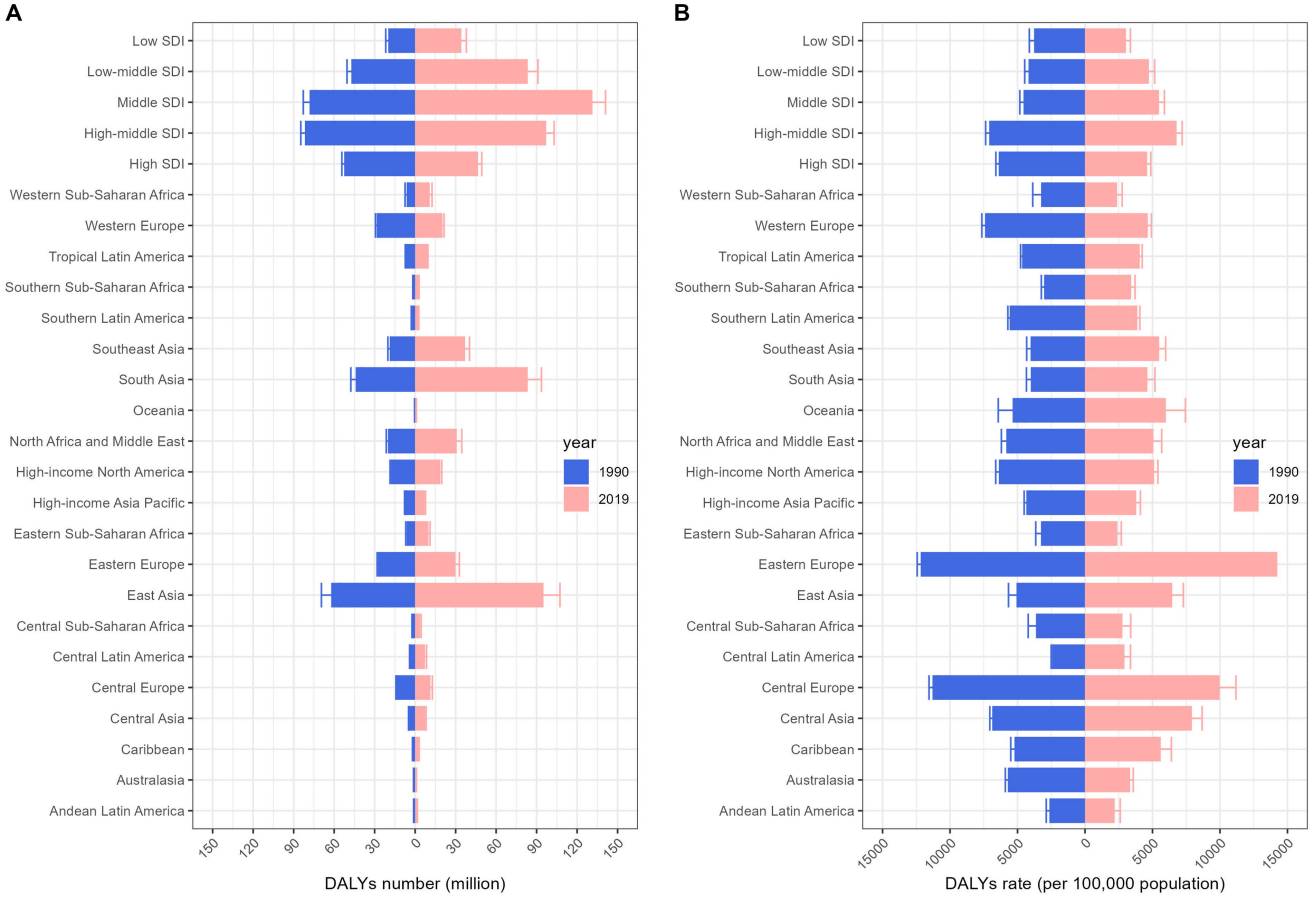
Change in burden of cardiovascular diseases by region, 1990 vs. 2019. Columns and error bars represent the central estimates and 95% uncertainty interval of DALYs cases **(A)** and DALYs rates **(B)** in the all-ages population, respectively. DALYs, disability-adjusted life-years; SDI, socio-demographic index.

**Figure 2:**
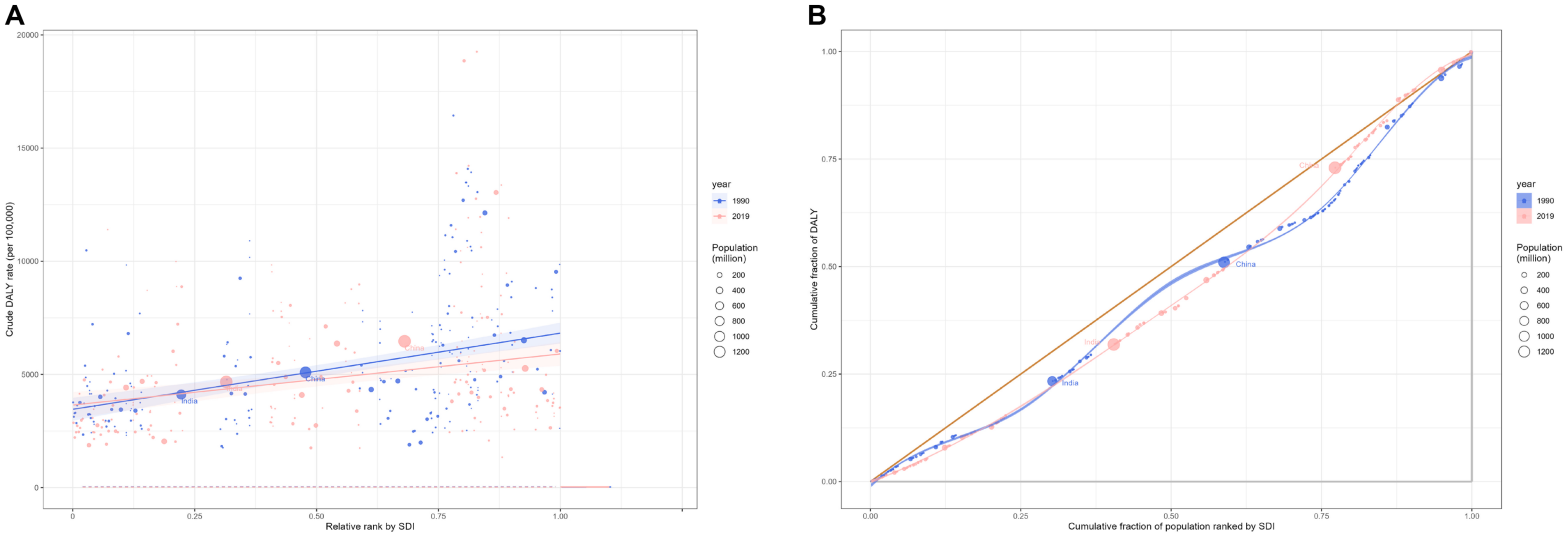
Health inequality regression curves **(A)** and concentration curves **(B)** for the DALYs of cardiovascular diseases worldwide, 1990 and 2019. DALYs, disability-adjusted life-year.

In terms of DALYs, the overall CVD burden for the all-age population in 2019 stood at 393.1 million (95% UI 367.8 to 417.2 million), marking a 40.5% rise from 1990’s 279.8 million (95% UI 267.7 to 291.1 million). The DALYs rate for 2019 was 5080.6 (95% UI 4753.2 to 5392.7) per 100,000 population, a 2.9% decrease from 1990’s 5230.8 (95% UI 5003.2 to 5440.3) per 100,000 population (Table 1). In 2019, the South Asian (83.4 million, 95% UI 74.1 to 93.6 million) and East Asian (95.1 million, 95% UI 83.3 to 107.3 million) regions bore the heaviest CVD burdens. Furthermore, the Middle and High-middle SDI regions experienced considerable CVD burdens. In developed regions, including Eastern Europe and Central Europe, the DALYs rate per 100,000 was most pronounced. From 1990 to 2019, most GBD regions witnessed a decline in the rate of DALYs (Figure 1). Observations revealed absolute and relative inequalities linked to SDI, with countries possessing higher SDI shouldering a disproportionately elevated burden (Figure 2). Additionally, the SII demonstrated the DALYs rate gap among nations from 3760.40 (95% CI: 3758.26 to 3756.53) in 1990 to 3400.38 (95% CI: 3398.64 to 3402.13) in 2019. The relative concentration index for 1990 and 2019 were 11.75 (95% CI: 11.42 to 12.09) and 10.96 (95% CI: 10.52 to 11.40), respectively (Table 2). Over time, the health inequality associated with SDI in CVD has decreased.

**Table 1:** Global burden of cardiovascular diseases and the specific causes in 1990 and 2019.

	DALYs cases			DALYs rates		
Diseases	1990 (million)*	2019 (million)*	Change (%)	1990 (/100,000)*	2019 (/100,000)*	Change (%)
Cardiovascular diseases	279.8 (267.7 ~ 291.1)	393.1 (367.8 ~ 417.2)	40.5	5230.8 (5003.2 ~ 5440.3)	5080.6 (4753.2 ~ 5392.7)	−2.9
Rheumatic heart disease	13.1 (11.9 ~ 14.6)	10.7 (9.2 ~ 12.1)	−18.9	246.1 (222.4 ~ 273.6)	137.9 (119.0 ~ 156.7)	−44.0
Ischemic heart disease	121.1 (116.4 ~ 125.6)	182.0 (170.2 ~ 193.5)	50.4	2263.0 (2175.0 ~ 2348.4)	2352.6 (2200.0 ~ 2500.9)	4.0
Stroke	108.2 (102.4 ~ 114.8)	143.2 (133.1 ~ 153.2)	32.3	2022.6 (1914.4 ~ 2145.8)	1851.2 (1720.1 ~ 1980.5)	−8.5
Hypertensive heart disease	13.9 (11.3 ~ 15.7)	21.5 (16.4 ~ 23.9)	54.2	260.6 (211.4 ~ 292.5)	278.0 (212.0 ~ 308.9)	6.7
Non-rheumatic valvular heart disease	1.7 (1.5 ~ 1.8)	2.8 (2.5 ~ 3.1)	67.6	31.2 (27.9 ~ 34.6)	36.1 (32.6 ~ 40.4)	7.3
Cardiomyopathy and myocarditis	7.1 (6.3 ~ 8.6)	9.1 (7.9 ~ 10.0)	29.4	132.0 (117.7 ~ 161.3)	118.1 (101.6 ~ 129.8)	−10.5
Atrial fibrillation and flutter	3.8 (3.0 ~ 4.8)	8.4 (6.7 ~ 10.5)	121.6	70.8 (55.3 ~ 90.3)	108.5 (86.5 ~ 136.2)	53.2
Aortic aneurysm	2.0 (1.8 ~ 2.2)	3.3 (3.1 ~ 3.5)	67.0	37.2 (34.0 ~ 41.0)	42.9 (40.2 ~ 45.6)	15.5
Peripheral artery disease	0.8 (0.5 ~ 1.2)	1.5 (1.0 ~ 2.4)	98.1	14.5 (9.1 ~ 22.0)	19.9 (13.0 ~ 30.6)	37.0
Endocarditis	1.1 (0.8 ~ 1.3)	1.7 (1.4 ~ 1.9)	54.2	20.9 (15.6 ~ 24.6)	22.2 (17.5 ~ 25.0)	6.6

*Central estimates with 95% uncertainty intervals. DALYs, disability-adjusted life-years.

**Table 2:** Summary measures for SDI-related inequalities in DALYs of cardiovascular diseases.

Diseases	Health inequality metrics	Year	Value	95% CI
Cardiovascular diseases	Slope index of inequality	1990	3760.40	(3758.26 ~ 3756.53)
		2019	3400.38	(3398.64 ~ 3402.13)
	Relative concentration index	1990	11.75	(11.42 ~ 12.09)
		2019	10.96	(10.52 ~ 11.40)
Rheumatic heart disease	Slope index of inequality	1990	−345.95	(−346.47 ~ −345.42)
		2019	−204.34	(−204.67 ~ −204.01)
	Relative concentration index	1990	−22.28	(−24.00 ~ −20.57)
		2019	−23.55	(−25.80 ~ −21.30)
Ischemic heart disease	Slope index of inequality	1990	2833.18	(2831.67 ~ 2834.69)
		2019	1560.28	(1559.07 ~ 1561.48)
	Relative concentration index	1990	20.17	(19.70 ~ 20.65)
		2019	10.85	(10.39 ~ 11.32)
Stroke	Slope index of inequality	1990	788.36	(787.04 ~ 789.69)
		2019	1237.48	(1236.40 ~ 1238.55)
	Relative concentration index	1990	6.40	(6.13 ~ 6.66)
		2019	10.94	(10.41 ~ 11.46)
Hypertensive heart disease	Slope index of inequality	1990	−82.07	(−82.56 ~ −81.59)
		2019	108.99	(108.57 ~ 109.40)
	Relative concentration index	1990	−5.16	(−5.60 ~ −4.71)
		2019	6.45	(5.95 ~ 6.95)
Non-rheumatic valvular heart disease	Slope index of inequality	1990	89.79	(89.55 ~ 30.03)
		2019	100.95	(100.74 ~ 101.16)
	Relative concentration index	1990	42.07	(40.32 ~ 43.82)
		2019	40.96	(39.30 ~ 42.62)
Cardiomyopathy and myocarditis	Slope index of inequality	1990	273.05	(272.62 ~ 273.47)
		2019	250.76	(250.42 ~ 251.09)
	Relative concentration index	1990	31.90	(29.22 ~ 34.58)
		2019	32.59	(30.49 ~ 34.69)
Atrial fibrillation and flutter	Slope index of inequality	1990	163.01	(162.69 ~ 163.34)
		2019	245.43	(245.09 ~ 245.76)
	Relative concentration index	1990	35.02	(32.62 ~ 37.42)
		2019	34.42	(31.97 ~ 36.87)
Aortic aneurysm	Slope index of inequality	1990	104.91	(104.65 ~ 105.17)
		2019	91.14	(90.94 ~ 91.35)
	Relative concentration index	1990	41.36	(39.48 ~ 43.25)
		2019	32.57	(31.25 ~ 33.89)
Peripheral artery disease	Slope index of inequality	1990	55.46	(55.27 ~ 55.66)
		2019	67.87	(67.70 ~ 68.05)
	Relative concentration index	1990	51.81	(44.71 ~ 58.91)
		2019	47.67	(40.72 ~ 54.61)
Endocarditis	Slope index of inequality	1990	−4.50	(−4.64 ~ −4.36)
		2019	16.00	(15.88 ~ 16.12)
	Relative concentration index	1990	−3.53	(−3.86 ~ −3.19)
		2019	11.72	(11.02 ~ 12.43)

DALYs, disability-adjusted life-years; SDI, sociodemographic index.

### 3.2 Rheumatic heart disease

**Figure 3:**
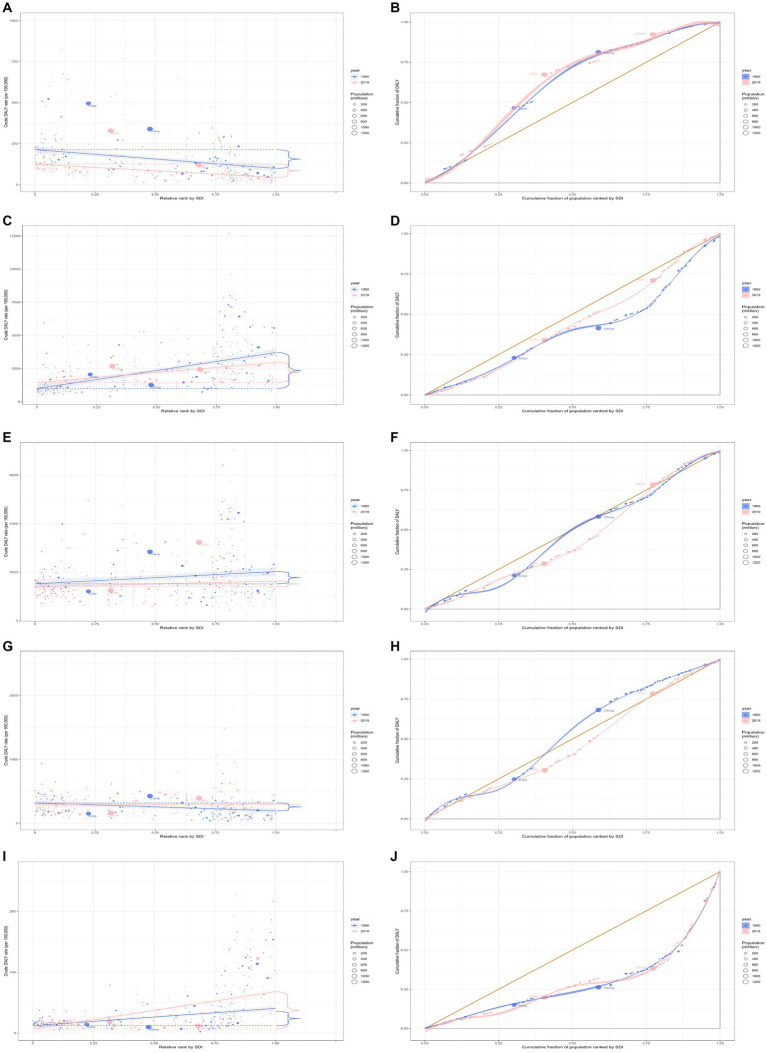
Health inequality regression curves and concentration curves for the DALYs of rheumatic heart disease **(A,B)**, ischemic heart disease **(C,D)**, stroke **(E,F)**, hypertensive heart disease **(G,H)**, and non-rheumatic valvular heart disease **(I,J)** worldwide, 1990 and 2019. DALYs, disability-adjusted life-years.

In 2019, the overall global burden of rheumatic heart disease across all ages was 10.7 million (95% UI 9.2 to 12.1), representing an 18.9% decline from 13.1 million (95% UI 11.9 to 14.6) in 1990. The DALYs rate in 2019 was 137.9 (95% UI 119.0 to 156.7) per 100,000 population, a 44.0% decrease from 246.1 (95% UI 222.4 to 273.6) per 100,000 population in 1990 (Table 1). In 2019, South Asia (5.8 million, 95% UI 4.6 to 6.8) and East Asia (1.8 million, 95% UI 1.5 to 2.0) bore the heaviest burden of rheumatic heart disease. Areas with low-middle and low SDI had a more significant burden. Globally, underdeveloped regions, including Oceania and South Asia, had the highest DALYs rate per 100,000 population. From 1990 to 2019, most GBD regions experienced a decline in the DALYs rate (Supplementary Figure S1). Regarding the burden of rheumatic heart disease, both absolute and relative inequalities associated with SDI were observed. Countries with a lower SDI bore a disproportionately higher burden (Figures 3A,B). Additionally, as indicated by the SII, the DALYs rate disparity among nations from-345.95 (95% CI: −346.47 to-345.42) in 1990 to-204.34 (95% CI: −204.67 to-204.01) in 2019. The relative concentration index indices for 1990 and 2019 were-22.28 (95% CI: −24.00 to −20.57) and −23.55 (95% CI: −25.80 to −21.30), respectively (Table 2). Over time, the health inequality associated with SDI in rheumatic heart disease has decreased.

### 3.3 Ischemic heart disease

In 2019, the global ischemic heart disease burden across all ages was 182.0 million (95% UI 170.2 to 193.5), an increase of 50.4% from 121.1 million (95% UI 116.4 to 125.6) in 1990 (Table 1). The burden in 2019 was more significant in Middle and High-middle SDI regions. Globally, developed regions, including Eastern Europe, Central Europe, and Central Asia, had the highest DALYs rate per 100,000 population (Supplementary Figure S2). Regarding the ischemic heart disease burden, absolute and relative inequalities related to SDI were observed, with countries with higher SDI bearing a higher burden (Figures 3C,D). Additionally, as indicated by the SII, the disparity in DALYs rate among nations has decreased from 2833.18 in 1990 (95% CI: 2831.67 to 2834.69) to 1560.28 in 2019 (95% CI: 1559.07 to 1561.48). The relative concentration index for 1990 and 2019 were 20.17 (95% CI: 19.70 to 20.65) and 10.85 (95% CI: 10.39 to 11.32), respectively (Table 2). Over time, the degree of health inequality associated with SDI in ischemic heart disease has decreased.

### 3.4 Stroke

In 2019, the overall global burden of stroke for the entire age population was 143.2 million (95% UI 133.1 to 153.2), marking a 32.3% increase from 108.2 million (95% UI 102.4 to 114.8) in 1990. The DALYs rate for 2019 was 1851.2 (95% UI 1720.1 to 1980.5) per 100,000, reflecting an 8.5% decrease from 2022.6 (95% UI 1914.4 to 2145.8) per 100,000 in 1990 (Table 1). The burden of stroke was notably significant in Middle and High-middle SDI regions in 2019. In developed regions, such as Eastern and Central Europe, the highest DALYs rate per 100,000 individuals was recorded (Supplementary Figure S3). A pronounced disparity, both absolute and relative, linked to SDI was observed in the burden of stroke, with countries of higher SDI bearing a disproportionately greater burden (Figures 3E,F). Additionally, as indicated by the SII, the difference in DALYs rate among countries expanded from 788.36 (95% CI: 787.04 to 789.69) in 1990 to 1237.48 (95% CI: 1236.40 to 1238.55) in 2019. The relative concentration index for 1990 and 2019 were 6.40 (95% CI: 6.13 to 6.66) and 10.94 (95% CI: 10.41 to 11.46), respectively (Table 2). Over time, the degree of health inequality associated with SDI in stroke has increased.

### 3.5 Hypertensive heart disease

In 2019, the overall global burden for hypertensive heart disease across all ages was 21.5 million (95% UI 16.4 to 23.9), an increase of 54.2% from 13.9 million (95% UI 11.3 to 15.7) in 1990 (Table 1). East Asia bore the heaviest burden of hypertensive heart disease, with 5.8 million (95% UI 4.1 to 6.7) in 2019. The burden was considerable in Middle SDI regions. Central Europe had the highest DALYs rate per 100,000. Between 1990 and 2019, the DALYs rate increased in most Global Burden of Disease (GBD) regions (Supplementary Figure S4). Disparities, both absolute and relative, associated with SDI were evident, with countries of lower SDI shouldering an exceedingly greater burden in 1990 and countries of higher SDI shouldering an exceedingly greater burden in 2019 (Figures 3G,H). Additionally, the SII revealed that the difference in the DALYs rate among the countries grew from-82.07 (95% CI: −82.56 to-81.59) in 1990 to 108.99 (95% CI: 108.57 to 109.40) in 2019. The relative concentration indices for these years were −5.16 (95% CI: −5.60 to-4.71) and 6.45 (95% CI: 5.95 to 6.95), respectively (Table 2). Over time, the degree of health inequality associated with SDI in hypertensive heart disease has increased.

### 3.6 Non-rheumatic valvular heart disease

In 2019, the overall global burden of non-rheumatic valvular heart disease among all age groups was estimated at 2.8 million (95% UI: 2.5 to 3.1 million), a 67.6% increase from the 1.7 million (95% UI: 1.5 to 1.8 million) reported in 1990 (Table 1). Western Europe was the most severely affected region in 2019, with 0.7 million cases (95% UI: 0.6 to 0.8 million). Regions with a high SDI bore a significant burden. Among developed regions such as Western Europe, High-income North America, and High-income Asia Pacific, the highest rates of DALYs per 100,000 population were observed. Between 1990 and 2019, there was an observed increase in rates of DALYs across most GBD regions (Supplementary Figure S5). Inequalities, both absolute and relative, linked with SDI were noted. High SDI countries bore a disproportionally larger burden (Figures 3I,J). Additionally, as indicated by the SII, the disparity of the DALYs rate among nations expanded from 89.79 (95% CI: 89.55 to 30.03) in 1990 to 100.95 (95% CI: 100.74 to 101.16) in 2019. The relative concentration index for 1990 and 2019 was 42.07 (95% CI: 40.32 to 43.82) and 40.96 (95% CI: 39.30 to 42.62), respectively (Table 2). Over time, the degree of health inequality associated with SDI in non-rheumatic valvular heart disease has increased.

### 3.7 Cardiomyopathy and myocarditis

**Figure 4:**
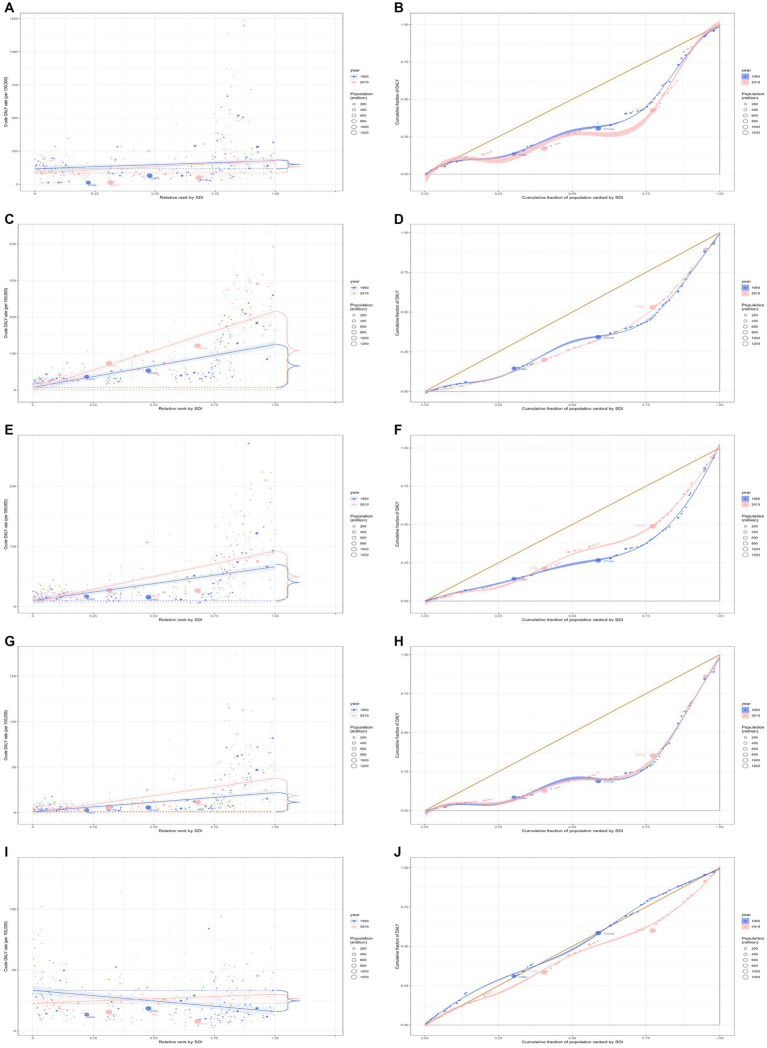
Health inequality regression curves and concentration curves for the DALYs of cardiomyopathy and myocarditis **(A,B)**, atrial fibrillation and flutter **(C,D)**, aortic aneurysm **(E,F)**, peripheral artery disease **(G,H)**, and endocarditis **(I,J)** worldwide, 1990 and 2019. DALYs, disability-adjusted life-years.

In 2019, the overall global burden for cardiomyopathy and myocarditis among all age groups was 9.1 million (95% UI: 7.9 to 10.0 million), marking a 29.4% increase from the 7.1 million (95% UI: 6.3 to 8.6 million) in 1990. The DALYs rate for 2019 was 118.1 (95% UI: 101.6 to 129.8) per 100,000, which is a 10.5% decline from 132.0 (95% UI: 117.7 to 161.3) per 100,000 in 1990 (Table 1). In 2019, Eastern Europe bore the most significant burden, with 0.7 million cases (95% UI: 0.6 to 0.8 million). Burdens were particularly high in regions with high and high-middle SDI. In more developed regions like Eastern Europe, the DALYs rates were the highest per 100,000 population (Supplementary Figure S6). Discrepancies associated with SDI were observed regarding the disease burden, with countries with a higher SDI boring a disproportionately larger burden (Figures 4A,B). Additionally, the SII indicated that the disparity in DALYs rates among countries reduced from 273.05 (95% CI: 272.62 to 273.47) in 1990 to 250.76 (95% CI: 250.42 to 251.09) in 2019. The relative concentration indexes for 1990 and 2019 were 31.90 (95% CI: 29.22 to 34.58) and 32.59 (95% CI: 30.49 to 34.69), respectively (Table 2). Over time, the degree of health inequality associated with SDI in cardiomyopathy and myocarditis disease has decreased.

### 3.8 Atrial fibrillation and flutter

In 2019, the overall global burden of atrial fibrillation and flutter for the entire age population stood at 8.4 million (95% UI 6.7 to 10.5 million), reflecting an increase of 121.6% from 3.8 million (95% UI 3.0 to 4.8 million) reported in 1990. The DALYs rate in 2019 was 108.5 per 100,000 (95% UI 86.5 to 136.2), marking a 53.2% increase from the 70.8 per 100,000 (95% UI 55.3 to 90.3) recorded in 1990 (Table 1). The East Asia region bore the heaviest burden, accounting for 1.8 million cases (95% UI 1.4 to 2.3 million). The burden was particularly pronounced in regions with high and high-middle SDI. In more developed regions, such as Europe, High-income North America, and Australasia, the highest rates of DALYs per 100,000 were recorded (Supplementary Figure S7). Notably, there were both absolute and relative discrepancies in burden associated with SDI, with higher SDI countries bearing a disproportionately greater burden (Figures 4C,D). Additionally, as depicted by the SII, the disparity in DALYs rate among countries escalated from 163.01 in 1990 (95% CI: 162.69 to 163.34) to 245.43 in 2019 (95% CI: 245.09 to 245.76). The relative concentration index values for 1990 and 2019 were 35.02 (95% CI: 32.62 to 37.42) and 34.42 (95% CI: 31.97 to 36.87), respectively (Table 2). Over time, the degree of health inequality associated with SDI in atrial fibrillation and flutter disease has increased.

### 3.9 Aortic aneurysm

In 2019, the overall global burden of aortic aneurysms across all age groups was 3.3 million (95% UI: 3.1 to 3.5 million), marking a 67.0% increase from 2.0 million (95% UI: 1.8 to 2.2 million) in 1990. The DALYs rate for 2019 was 42.9 per 100,000 (95% UI: 40.2 to 45.6), an increase of 15.5% from 37.2 per 100,000 (95% UI: 34.0 to 41.0) in 1990 (Table 1). East Asia bore the heaviest burden of aortic aneurysm, with 0.4 million cases (95% UI: 0.3 to 0.5 million). High and high-middle SDI regions showed a substantial burden. In developed regions such as Europe, High-income Asia Pacific, and America, the DALYs rate per 100,000 population was the highest (Supplementary Figure S8). There was evident absolute and relative inequality related to SDI, with nations having higher SDI shouldering disproportionately greater burdens (Figures 4E,F). Additionally, as demonstrated by the SII, the DALYs rate difference among nations decreased from 104.91 in 1990 (95% CI: 104.65 to 105.17) to 91.14 in 2019 (95% CI: 90.94 to 91.35). The relative concentration indexes for 1990 and 2019 were 41.36 (95% CI: 39.48 to 43.25) and 32.57 (95% CI: 31.25 to 33.89), respectively (Table 2). Over time, the degree of health inequality associated with SDI in aortic aneurysms has decreased.

### 3.10 Peripheral artery disease

In 2019, the overall global burden of peripheral artery disease across all age groups was 1.5 million (95% UI: 1.0 to 2.4 million), an escalation of 98.1% from the 0.8 million (95% UI: 0.5 to 1.2 million) reported in 1990. The DALYs rate in 2019 was 19.9 per 100,000 (95% UI: 13.0 to 30.6), a 37.0% rise from the 14.5 per 100,000 (95% UI: 9.1 to 22.0) in 1990 (Table 1). Western Europe was the region most affected by peripheral artery disease, with 0.3 million cases (95% UI: 0.2 to 0.5 million). High and high-middle SDI regions displayed a pronounced burden. In developed areas, including Europe, High-income North America, and Australasia, the DALYs rate per 100,000 was the highest (Supplementary Figure S9). Observations indicated absolute and relative inequality tied to SDI, with nations with a higher SDI incurring a disproportionately greater burden (Figures 4G,H). Additionally, as the SII reflects, the DALYs rate disparity among nations increased from 55.46 in 1990 (95% CI: 55.27 to 55.66) to 67.87 in 2019 (95% CI: 67.70 to 68.05). The relative concentration indexes for 1990 and 2019 were 51.81 (95% CI: 44.71 to 58.91) and 47.67 (95% CI: 40.72 to 54.61), respectively (Table 2). Over time, the degree of health inequality associated with SDI in peripheral artery disease has increased.

### 3.11 Endocarditis

For 2019, the overall global burden of endocarditis across all age groups was 1.7 million (95% UI 1.4 to 1.9 million), a rise of 54.2% from the 1.1 million (95% UI 0.8 to 1.3 million) observed in 1990. The DALYs rate in 2019 was 22.2 per 100,000 (95% UI 17.5 to 25.0 per 100,000), marking an increase of 6.6% from 20.9 per 100,000 (95% UI 15.6 to 24.6 per 100,000) in 1990 (Table 1). These regions most heavily burdened by endocarditis were South Asia and Southeast Asia, each reporting 0.3 million cases (95% UI 0.2 to 0.4 million). Furthermore, regions with high and high-middle Socio-Demographic Indexes (SDI) exhibited a pronounced burden of endocarditis. For developed regions, such as Western Europe, High-income North America, and Southern Latin America, the DALYs rate per 100,000 was notably high (Supplementary Figure S10). Disparities in absolute and relative inequalities linked with SDI were observed in the burden, with countries having lower SDIs shouldering disproportionately elevated burdens in 1990 and countries having higher SDIs shouldering disproportionately elevated burdens in 2019 (Figures 4I,J). As indicated by SII, the gap in the DALYs rate among nations expanded from-4.50 in 1990 (95% CI: −4.64 to-4.36) to 16.00 in 2019 (95% CI: 15.88 to 16.12). Relative concentration indices for 1990 and 2019 stood at −3.53 (95% CI: −3.86 to −3.19) and 11.72 (95% CI: 11.02 to 12.43), respectively (Table 2). Over time, the health inequality associated with SDI in endocarditis has increased.

In addition, the level of transnational inequality related to the level of sociodemographic development over 30 years for total cardiovascular disease and ten specific types of cardiovascular disease is shown in Supplementary Tables S3, S4.

## 4 Discussion

Although some research has reported on the health inequality of CVD, they have used national data (26, 27) or focused on the impact of risk factors on CVD (28–31). Therefore, in this research, we utilized a broader set of data from the 2019 Global Burden of Disease (GBD), spanning a longer observation period (1990–2019) and covering an extensive geographical scope (globally across 204 countries). Based on this data, we described the burden of CVD and reported changes in transnational socioeconomic inequality due to CVD between 1990 and 2019. Our findings indicate that nations with lower levels of societal development may shoulder the relatively burden of rheumatic heart disease. In contrast, countries with higher societal development levels may bear the relatively burden of nine other CVDs. The level of health inequality linked with SDI for conditions such as rheumatic heart disease, ischemic heart disease, cardiomyopathy and myocarditis, and aortic aneurysm has declined over time.

It is generally believed that individuals residing in countries with higher SDIs potentially have more access to and benefit from higher quality health and medical services, possibly incurring a reduced disease burden. However, this study quantified inequality based on different demographic, socioeconomic, or geographical characteristics, which aids in comparing health disparities across different periods, backgrounds, and indicators. Several factors may account for the atypical association observed between the burden of cardiovascular diseases and the level of social development. The global increase in CVD-related DALYs can be attributed to population growth and aging, which are the primary drivers of the overall increase in CVD burden (2). It is estimated that the global population has grown from 5.35 billion (95% UI 5.24 to 5.46 billion) in 1990 to 7.74 billion (95% UI 7.48 to 7.99 billion) in 2019 (18). By 2030, the global older adult population is projected to exceed 1.4 billion (32).

Atherosclerotic disease, within the spectrum of cardiovascular diseases, has traditionally been considered a condition prevalent in affluent nations, aligning with the epidemiological transition concept. High systolic blood pressure, high body mass index, and high low-density lipoprotein cholesterol are regarded as the three most important factors for cardiovascular disease DALY (2). Compared to low and middle-SDI countries/regions, young people in high-SDI countries/regions report higher frequencies of high stress, abdominal obesity, smoking, and alcohol consumption (19). Recent studies in the United States, European countries, and China have observed an increasing trend in stroke incidence among middle-aged individuals (33–35). Similarly, this trend may reflect an increased exposure to certain stroke risk factors in most countries, such as high blood pressure, high BMI, and elevated fasting blood glucose (36). In the United States, a concerning trend observed in recent years (2017–2018) is a decreasing hypertension awareness among the population with controlled blood pressure (37). Furthermore, the decline in stroke incidence rates in most countries is insufficient to offset the population growth and aging, resulting in an overall increase in stroke disability over time. A study on income groups by the World Bank found that the likelihood of new cases of subarachnoid hemorrhage in the high-income group is more than twice that of the combined low-income to middle-high-income group, and the increased risk of cerebral hemorrhage in high-income countries may be related to the high relative clinical significance and population attributable risk of hypertension in these countries (38).In addition, approximately 70% of the global burden of peripheral artery disease (PAD) is attributed to modifiable risk factors, highlighting the potential of public health measures to mitigate the burden of PAD by targeting these risk factors (39). The increasing SDI is associated with an increased burden of PAD, potentially due to elevated metabolic pressures, such as hypertension and blood glucose, in high-income countries (40). Studies have indicated a U-shaped pattern in the DALY and mortality rates of PAD, with the highest DALY and mortality rates reported in countries with the highest SDI and income levels (39). This pattern may suggest that the burden of PAD in countries with low SDI and income is disproportionate to the prevalence, indicating inadequate management of the PAD burden in these countries. Considering the lower prevalence of PAD in low SDI and low-income countries, mild or asymptomatic PAD may be a result of resource limitations (41). Additionally, factors associated with socioeconomic underdevelopment, such as limited access to care, inadequate quality of care, and unfavorable conditions for lifestyle changes, may contribute to a more severe burden of the disease (42). Therefore, timely diagnosis and appropriate management of PAD in resource-constrained regions must be emphasized in the international community, along with the exploration of systematic-level differences in managing cardiovascular risk factors.

For atrial fibrillation (AF), social factors significantly impact changes in DALYs. Studies have shown lower incidence rates in low SDI regions, suggesting a higher proportion of undetected AF cases, particularly among individuals with paroxysmal or asymptomatic AF (43). These disparities in detection and diagnosis may be attributed to the unequal distribution of healthcare resources and strategies in different socio-economic environments. At the same time, cardiomyopathy is a major public health issue in Eastern Europe (44). For example, binge drinking patterns such as alcohol, despite restrictive alcohol policies in these countries, have helped to reduce alcohol-related mortality, but the burden remains very high, consistent with our study (45). Future strategies are needed in Eastern European countries to strengthen the management of cardiomyopathy to address health challenges. From 1990 to 2019, with the advancement of medical technology, the increase in pathogenic bacteria and the proliferation of implanted cardiac electronic devices have likely contributed to the increased burden of infective endocarditis in high-income countries (46). A recent review of key cardiac and non-cardiac risk factors includes rheumatic heart disease, prosthetic valves, intravenous drug use, and cardiac electronic devices (47). In the United States, approximately 50% of these cases are healthcare-associated and continue to rise, particularly with nosocomial endocarditis, including prosthetic valve-related endocarditis, intravenous drug use-related endocarditis, and cardiac electronic device-related endocarditis (48). Similar trends can also be observed in other developed countries, such as Spain and Italy (49, 50). Additionally, the significant variation in the burden of endocarditis may be attributed to the increasing burden of countries with high SDI levels due to drugs and an aging population (51).

The Social Demographic Index primarily comprises the education years, the average income level, and the total fertility rate. Therefore, due to the different transitions in population growth and aging in different countries, regions should devise corresponding strategies according to their circumstances. The high exposure to risk factors for CVD among young people in developed countries – such as obesity, physical inactivity, poor diet, and psychosocial stresses – plays a crucial role (6, 8, 52, 53). Furthermore, while mortality rates of CVD have decreased in the past 50 years in high-income countries (54), the extended life expectancy and an aging population mean many patients face ongoing out-of-pocket costs during their long-term healthcare journeys, which can potentially plunge their families into poverty or even lead to catastrophic healthcare expenses, exacerbating health inequities related to CVD (2, 55). Moreover, the global inequality in burden for diseases such as rheumatic heart disease, ischemic heart disease, cardiomyopathy and myocarditis, and aortic aneurysms has steadily diminished over the past 30 years. This shift is attributed to nations recognizing these diseases as major public health issues and directing substantial resources toward monitoring and managing risk factors like alcohol consumption, smoking, and hypertension (56). Additionally, the widespread usage of medicines like aspirin, statins, diuretics, and β-blockers, advanced medical technologies, and public health education campaigns have likely diminished these health inequities (56). Rheumatic heart disease is caused by an autoimmune response to Group A *Streptococcal* infections, which leads to long-term damage to the heart valves (57). Those living in impoverished areas bear a higher burden due to poor sanitary conditions, repeated infections from exposure to harmful pathogens, and lack of access to professional guidelines and standardized treatment with medicines like penicillin (58). Most countries with higher societal development levels have, in the past 30 years, seen increasing urbanization and modernization, leading to heightened exposures to harmful substances such as light and chemical pollutants, escalating urban carbon emissions, and deteriorating air quality, which has spurred the incidence of CVD (59). While significant progress has been made in preventing and managing CVD, they continue to impose substantial health and economic burdens on individuals, healthcare systems, and societies. As time progresses, the inequality in the CVD burden associated with SDI has significantly increased in many countries, suggesting that, over the past 30 years, with the increase in societal development levels, investments in cardiovascular prevention, management, and treatment might have been insufficient, and inequality may persist. This result underscores the need to address cardiovascular disparities primarily through economic, dietary, and lifestyle interventions. Adopting a plant-based, low-fat diet supplemented with high-quality protein intake can reduce low-density lipoprotein cholesterol levels, thereby mitigating the onset of CVD (60, 61). In high-income areas, refining secondary prevention and treatment should be prioritized in policy-making, enhancing mitigation of risk factors such as obesity and ensuring increased access to high-quality treatment and care. In low-income areas, it is necessary to enhance people’s awareness of CVD and improve healthcare workers’ diagnostic capabilities to emphasize the early detection and prevention of CVD.

Several limitations should be noted. Firstly, due to the imperfections in the healthcare systems of underdeveloped countries, misdiagnosis and missed diagnoses could occur in GBD studies. These could pose challenges to the accurate assessment of CVD. Secondly, to overcome the unbalanced quality caused by massive amounts of original data from different countries, the GBD collaborators adopted efficient data cleaning methods and advanced statistical modeling. However, this could lead to an over-reliance on modeled data in GBD studies and a failure to consider sociocultural and ethnic differences. Additionally, diagnostic capabilities for CVD might evolve with societal and technological advancements, and people’s understanding of diseases and acceptance of health education are changing.

## 5 Conclusion

In summary, leveraging global data from the 2019 GBD, our research investigates transnational inequality for overall CVD and ten specific CVDs. We found that countries with higher SDI may bear a relatively higher burden of CVD, except for rheumatic heart disease. Over time, the level of health inequality linked with SDI for conditions like rheumatic heart disease, ischemic heart disease, cardiomyopathy and myocarditis, and aortic aneurysm has diminished, and for other CVDs, the level of health inequality linked with SDI has increased. However, in low- and middle-income countries, the large population, the serious underreporting and inadequate healthcare systems make this issue even more complex. There is an urgent need for further research into determinants of the CVD burden and to establish effective strategies for the management of CVD in countries with higher levels of societal development and strengthen early diagnosis and prevention in low- and middle-income countries.

## Data availability statement

The original contributions presented in the study are included in the article/Supplementary material, further inquiries can be directed to the corresponding authors.

## Ethics statement

The studies involving humans were approved by Ethics Committee of the Second People’s Hospital of Hefei. The studies were conducted in accordance with the local legislation and institutional requirements. Written informed consent for participation was not required from the participants or the participants’ legal guardians/next of kin in accordance with the national legislation and institutional requirements.

## Author contributions

BH: Conceptualization, Data curation, Formal analysis, Investigation, Methodology, Project administration, Resources, Software, Supervision, Validation, Visualization, Writing – original draft, Writing – review & editing. JF: Supervision, Writing – review & editing. YW: Supervision, Writing – review & editing. LH: Supervision, Writing – review & editing. YF: Supervision, Writing – review & editing.

## Footnotes

^1^http://ghdx.healthdata.org/gbd-results-tool
