# Supplementary material for: Transnational inequities in cardiovascular diseases from 1990 to 2019: exploration based on the global burden of disease study 2019
Source: Front Public Health. 2024 Apr 3;12:1322574. doi: 10.3389/fpubh.2024.1322574 (PMC11021694; doi:10.3389/fpubh.2024.1322574)
Supplement: Supplementary file 1 [file Data_Sheet_1.docx]

***Supplementary Material***

**Transnational Inequities in Cardiovascular Diseases from 1990 to 2019：exploration based on the Global Burden of Disease Study 2019**

**Ben Hu*, Jun Feng, Yuhui Wang, Linlin Hou, Yinguang Fan**

**Correspondence:** Linlin Hou: [2245012179@stu.ahmu.edu.cn](mailto:2245012179@stu.ahmu.edu.cn)

Yinguang Fan: fanyinguang@163.com

**Supplementary Figures and Tables**

Table S1. International Classification of Disease 10th revision (ICD-10) codes for CVD categories according to GBD 2019 study.

| CVD category | ICD-10 Codes |
| --- | --- |
| Ischemic Heart Disease | I20-I25.9 |
| Stroke | G45-G46.8, I63-I63.9, I65-I66.9, I67.2-I67.3, I67.5-I67.6, I69.3, I60-I62.9, I67.0-I67.1, I68.1-I68.2, I69.0-I69.2 |
| Atrial Fibrillation and Flutter | I48-I48.9 |
| Rheumatic Heart Disease | I01-I01.9, I02.0, I05-I09.9 |
| Non-rheumatic valvular heart disease | I34-137.9 |
| Hypertensive Heart Disease | I11-I11.9 |
| Cardiomyopathy and myocarditis | B33.2, I40-I41.9, I42.1-I42.8, I43-I43.9, I51.4 |
| Aortic Aneurysm | I71-I71.9 |
| Peripheral Artery Diseases | I70.2-I70.8, I73-I73.9 |
| Endocarditis | I33-I33.9, I38-I39.9 |

Table S2. Population and economic status of country and territories in 1990 and 2019.

|  | **1990** | | **2019** | |
| --- | --- | --- | --- | --- |
|  | **Population a** | **SDI ^b^** | **Population a** | **SDI ^b^** |
| Afghanistan | 11·42 (9·8 to 12·98) | 0·187 | 38·28 (26·16 to 50·47) | 0·343 |
| Albania | 3·31 (3·05 to 3·57) | 0·54 | 2·72 (2·42 to 3·02) | 0·681 |
| Algeria | 25·28 (23·11 to 27·32) | 0·436 | 41·85 (36·02 to 47·46) | 0·652 |
| American Samoa | 0·05 (0·05 to 0·05) | 0·606 | 0·06 (0·05 to 0·06) | 0·712 |
| Andorra | 0·05 (0·05 to 0·05) | 0·834 | 0·08 (0·08 to 0·09) | 0·894 |
| Angola | 10·32 (8·41 to 12·4) | 0·238 | 30·14 (27·05 to 33·12) | 0·47 |
| Antigua and Barbuda | 0·06 (0·06 to 0·07) | 0·579 | 0·09 (0·08 to 0·1) | 0·743 |
| Argentina | 33·12 (30·78 to 35·43) | 0·581 | 45·12 (39·51 to 51·07) | 0·708 |
| Armenia | 3·41 (3·16 to 3·68) | 0·536 | 3·02 (2·65 to 3·39) | 0·689 |
| Australia | 16·86 (15·61 to 18·09) | 0·738 | 24·57 (22·51 to 26·78) | 0·839 |
| Austria | 7·77 (7·23 to 8·33) | 0·753 | 8·92 (8·17 to 9·67) | 0·849 |
| Azerbaijan | 7·33 (6·81 to 7·88) | 0·576 | 10·28 (8·95 to 11·64) | 0·683 |
| Bahamas | 0·26 (0·24 to 0·27) | 0·692 | 0·38 (0·33 to 0·42) | 0·796 |
| Bahrain | 0·51 (0·47 to 0·55) | 0·553 | 1·44 (1·25 to 1·61) | 0·751 |
| Bangladesh | 109·05 (101·36 to 117·15) | 0·267 | 159·26 (141·2 to 177·85) | 0·483 |
| Barbados | 0·25 (0·24 to 0·27) | 0·649 | 0·3 (0·26 to 0·33) | 0·742 |
| Belarus | 10·47 (9·67 to 11·26) | 0·591 | 9·5 (8·35 to 10·68) | 0·745 |
| Belgium | 9·98 (9·17 to 10·74) | 0·746 | 11·42 (10·54 to 12·32) | 0·851 |
| Belize | 0·19 (0·17 to 0·2) | 0·428 | 0·41 (0·36 to 0·46) | 0·603 |
| Benin | 4·85 (4·47 to 5·25) | 0·209 | 12·67 (11·32 to 13·98) | 0·352 |
| Bermuda | 0·06 (0·05 to 0·06) | 0·685 | 0·06 (0·06 to 0·07) | 0·813 |
| Bhutan | 0·61 (0·52 to 0·71) | 0·228 | 0·75 (0·7 to 0·82) | 0·455 |
| Bolivia (Plurinational State of) | 6·42 (5·91 to 6·91) | 0·412 | 12·01 (10·64 to 13·42) | 0·566 |
| Bosnia and Herzegovina | 4·54 (4·22 to 4·86) | 0·533 | 3·3 (2·95 to 3·65) | 0·718 |
| Botswana | 1·3 (1·2 to 1·39) | 0·431 | 2·34 (2·08 to 2·61) | 0·634 |
| Brazil | 148·84 (138·23 to 159·33) | 0·487 | 216·66 (189·88 to 242·5) | 0·64 |
| Brunei Darussalam | 0·26 (0·24 to 0·28) | 0·676 | 0·44 (0·38 to 0·49) | 0·823 |
| Bulgaria | 8·68 (7·97 to 9·39) | 0·631 | 6·93 (6·36 to 7·55) | 0·764 |
| Burkina Faso | 9·56 (8·61 to 10·52) | 0·125 | 22·69 (19·38 to 26·17) | 0·257 |
| Burundi | 5·57 (5·2 to 5·94) | 0·198 | 11·93 (10·3 to 13·53) | 0·284 |
| Cabo Verde | 0·35 (0·33 to 0·38) | 0·292 | 0·56 (0·49 to 0·63) | 0·525 |
| Cambodia | 10·38 (9·19 to 11·62) | 0·266 | 16·6 (14·21 to 18·87) | 0·469 |
| Cameroon | 10·39 (9·49 to 11·32) | 0·313 | 29·1 (24·78 to 33·6) | 0·49 |
| Canada | 27·26 (25·2 to 29·42) | 0·790 | 36·52 (33·33 to 39·6) | 0·873 |
| Central African Republic | 2·74 (2·53 to 2·98) | 0·186 | 5·3 (4·46 to 6·19) | 0·274 |
| Chad | 6·02 (5·5 to 6·55) | 0·108 | 16·4 (14·33 to 18·68) | 0·238 |
| Chile | 13·28 (12·24 to 14·35) | 0·592 | 18·2 (16·75 to 19·62) | 0·759 |
| China | 1183·68 (1103·09 to 1271·61) | 0·433 | 1422·35 (1239·3 to 1597·06) | 0·686 |
| Colombia | 32·55 (29·72 to 35·41) | 0·478 | 47·78 (44·17 to 51·54) | 0·633 |
| Comoros | 0·47 (0·43 to 0·5) | 0·274 | 0·71 (0·59 to 0·84) | 0·455 |
| Congo | 2·44 (2·17 to 2·7) | 0·364 | 5·27 (4·51 to 6·01) | 0·568 |
| Cook Islands | 0·02 (0·02 to 0·02) | 0·625 | 0·02 (0·02 to 0·02) | 0·764 |
| Costa Rica | 3·04 (2·73 to 3·36) | 0·532 | 4·72 (4·16 to 5·27) | 0·68 |
| Croatia | 4·9 (4·53 to 5·28) | 0·68 | 4·25 (3·75 to 4·76) | 0·794 |
| Cuba | 10·83 (9·51 to 12·09) | 0·578 | 11·36 (10·09 to 12·74) | 0·668 |
| Cyprus | 0·78 (0·72 to 0·84) | 0·662 | 1·31 (1·16 to 1·48) | 0·841 |
| Czechia | 10·3 (9·47 to 11·07) | 0·688 | 10·64 (9·78 to 11·5) | 0·828 |
| CÃ´te d'Ivoire | 12·23 (11·19 to 13·24) | 0·256 | 26·17 (23·57 to 28·87) | 0·408 |
| Democratic People's Republic of  Korea | 21·05 (19·26 to 22·82) | 0·431 | 26·23 (22·63 to 29·91) | 0·558 |
| Democratic Republic of the Congo | 38·59 (34·64 to 42·63) | 0·26 | 87·67 (61·75 to 112·59) | 0·382 |
| Denmark | 5·14 (5·11 to 5·18) | 0·806 | 5·8 (5·33 to 6·26) | 0·89 |
| Djibouti | 0·49 (0·43 to 0·54) | 0·275 | 1·2 (1·05 to 1·36) | 0·459 |
| Dominica | 0·07 (0·07 to 0·08) | 0·579 | 0·07 (0·06 to 0·08) | 0·729 |
| Dominican Republic | 7·2 (6·58 to 7·81) | 0·425 | 10·88 (9·63 to 12·28) | 0·592 |
| Ecuador | 10·03 (9·32 to 10·7) | 0·503 | 17·59 (15·4 to 19·75) | 0·64 |
| Egypt | 55·7 (50·56 to 60·91) | 0·403 | 99·07 (90·57 to 107·52) | 0·658 |
| El Salvador | 5·27 (4·85 to 5·68) | 0·39 | 6·26 (5·39 to 7·1) | 0·573 |
| Equatorial Guinea | 0·43 (0·38 to 0·48) | 0·208 | 1·42 (1·29 to 1·55) | 0·685 |
| Eritrea | 3 (2·67 to 3·32) | 0·198 | 6·71 (4·78 to 8·6) | 0·396 |
| Estonia | 1·57 (1·56 to 1·58) | 0·665 | 1·31 (1·2 to 1·42) | 0·835 |
| Eswatini | 0·81 (0·73 to 0·88) | 0·392 | 1·14 (1·05 to 1·23) | 0·577 |
| Ethiopia | 51·39 (46·66 to 56·07) | 0·144 | 107·59 (92·02 to 122·78) | 0·343 |
| Fiji | 0·76 (0·69 to 0·83) | 0·527 | 0·91 (0·84 to 0·98) | 0·664 |
| Finland | 5·01 (4·98 to 5·04) | 0·757 | 5·53 (5·09 to 5·99) | 0·856 |
| France | 57·77 (53·65 to 61·44) | 0·738 | 66·2 (60·09 to 72·43) | 0·834 |
| Gabon | 0·99 (0·91 to 1·09) | 0·388 | 1·75 (1·57 to 1·94) | 0·656 |
| Gambia | 0·99 (0·91 to 1·08) | 0·218 | 2·25 (2·03 to 2·48) | 0·399 |
| Georgia | 5·51 (5·1 to 5·92) | 0·654 | 3·66 (3·31 to 4·04) | 0·702 |
| Germany | 79·94 (79·37 to 80·54) | 0·819 | 84·91 (77·69 to 92·22) | 0·898 |
| Ghana | 15·02 (13·4 to 16·6) | 0·355 | 31·54 (27·45 to 35·19) | 0·557 |
| Greece | 10·39 (9·62 to 11·17) | 0·682 | 10·34 (9·07 to 11·49) | 0·794 |
| Greenland | 0·06 (0·06 to 0·06) | 0·655 | 0·06 (0·05 to 0·06) | 0·761 |
| Grenada | 0·09 (0·08 to 0·09) | 0·463 | 0·1 (0·09 to 0·12) | 0·669 |
| Guam | 0·14 (0·13 to 0·15) | 0·693 | 0·17 (0·15 to 0·19) | 0·813 |
| Guatemala | 7·97 (7·18 to 8·71) | 0·315 | 17·78 (14·65 to 20·93) | 0·526 |
| Guinea | 6·19 (5·51 to 6·86) | 0·175 | 12·64 (11·37 to 13·95) | 0·325 |
| Guinea-Bissau | 1·01 (0·93 to 1·08) | 0·2 | 1·9 (1·67 to 2·15) | 0·355 |
| Guyana | 0·77 (0·71 to 0·82) | 0·452 | 0·77 (0·68 to 0·86) | 0·618 |
| Haiti | 6·36 (5·58 to 7·12) | 0·307 | 12·4 (10·37 to 14·71) | 0·432 |
| Honduras | 4·71 (4·34 to 5·1) | 0·33 | 9·81 (8·82 to 10·83) | 0·496 |
| Hungary | 10·39 (9·64 to 11·13) | 0·659 | 9·67 (8·52 to 10·79) | 0·791 |
| Iceland | 0·25 (0·25 to 0·26) | 0·764 | 0·34 (0·32 to 0·37) | 0·869 |
| India | 855·59 (791·72 to 918·89) | 0·327 | 1390·71 (1237·77 to 1558·77) | 0·566 |
| Indonesia | 185·38 (172·86 to 197·99) | 0·452 | 259·47 (226·84 to 292) | 0·66 |
| Iran (Islamic Republic of) | 58·54 (53·28 to 63·54) | 0·404 | 84·3 (77·33 to 91·94) | 0·67 |
| Iraq | 17·6 (15·98 to 19·18) | 0·392 | 42·12 (31·43 to 52·98) | 0·671 |
| Ireland | 3·6 (3·33 to 3·86) | 0·73 | 4·91 (4·48 to 5·36) | 0·867 |
| Israel | 4·96 (4·47 to 5·45) | 0·717 | 9·31 (8·16 to 10·55) | 0·803 |
| Italy | 56·8 (52·81 to 60·69) | 0·712 | 60·31 (55·36 to 64·98) | 0·801 |
| Jamaica | 2·36 (2·18 to 2·54) | 0·542 | 2·81 (2·48 to 3·13) | 0·684 |
| Japan | 125·87 (117·09 to 134·21) | 0·791 | 127·79 (115·77 to 139·88) | 0·87 |
| Jordan | 3·77 (3·43 to 4·13) | 0·52 | 11·64 (10·59 to 12·68) | 0·731 |
| Kazakhstan | 16·37 (15·09 to 17·53) | 0·602 | 18·39 (16·79 to 19·92) | 0·723 |
| Kenya | 23·19 (21·58 to 24·92) | 0·333 | 50·23 (43·65 to 56·75) | 0·508 |
| Kiribati | 0·07 (0·07 to 0·08) | 0·425 | 0·12 (0·11 to 0·13) | 0·527 |
| Kuwait | 1·76 (1·59 to 1·93) | 0·655 | 4·43 (3·93 to 4·93) | 0·851 |
| Kyrgyzstan | 4·46 (4·12 to 4·81) | 0·532 | 6·54 (5·7 to 7·32) | 0·596 |
| Lao People's Democratic Republic | 4·15 (3·72 to 4·56) | 0·268 | 7·16 (6·47 to 7·83) | 0·49 |
| Latvia | 2·66 (2·46 to 2·86) | 0·675 | 1·92 (1·76 to 2·07) | 0·82 |
| Lebanon | 3·28 (2·73 to 3·82) | 0·462 | 5·18 (4·46 to 5·93) | 0·708 |
| Lesotho | 1·81 (1·65 to 1·97) | 0·321 | 2·09 (1·91 to 2·27) | 0·507 |
| Liberia | 1·96 (1·75 to 2·17) | 0·221 | 4·79 (4·13 to 5·42) | 0·37 |
| Libya | 4·24 (3·82 to 4·67) | 0·405 | 6·74 (5·71 to 7·67) | 0·709 |
| Lithuania | 3·67 (3·4 to 3·95) | 0·67 | 2·79 (2·57 to 3·03) | 0·843 |
| Luxembourg | 0·38 (0·35 to 0·41) | 0·815 | 0·62 (0·57 to 0·67) | 0·895 |
| Madagascar | 11·95 (10·9 to 12·98) | 0·265 | 26·69 (20·37 to 32·84) | 0·396 |
| Malawi | 9·56 (8·75 to 10·35) | 0·213 | 18·44 (17·15 to 19·75) | 0·384 |
| Malaysia | 17·66 (16·28 to 18·99) | 0·542 | 31·3 (27·34 to 35·19) | 0·737 |
| Maldives | 0·22 (0·21 to 0·24) | 0·303 | 0·5 (0·45 to 0·55) | 0·562 |
| Mali | 8·67 (7·92 to 9·47) | 0·126 | 21·92 (19·13 to 24·87) | 0·263 |
| Malta | 0·37 (0·33 to 0·41) | 0·666 | 0·44 (0·39 to 0·49) | 0·801 |
| Marshall Islands | 0·05 (0·04 to 0·05) | 0·398 | 0·06 (0·05 to 0·06) | 0·544 |
| Mauritania | 2·07 (1·9 to 2·24) | 0·308 | 4·01 (3·56 to 4·44) | 0·496 |
| Mauritius | 1·1 (1·03 to 1·18) | 0·527 | 1·28 (1·11 to 1·44) | 0·705 |
| Mexico | 85·49 (79·47 to 91·26) | 0·507 | 124·94 (108·61 to 140·63) | 0·649 |
| Micronesia (Federated States of) | 0·1 (0·09 to 0·12) | 0·447 | 0·1 (0·09 to 0·11) | 0·58 |
| Monaco | 0·03 (0·03 to 0·03) | 0·834 | 0·04 (0·03 to 0·04) | 0·902 |
| Mongolia | 2·15 (2 to 2·32) | 0·465 | 3·39 (2·98 to 3·8) | 0·606 |
| Montenegro | 0·63 (0·58 to 0·67) | 0·701 | 0·62 (0·55 to 0·7) | 0·791 |
| Morocco | 25·3 (22·97 to 27·68) | 0·347 | 35·95 (32·34 to 39·43) | 0·548 |
| Mozambique | 13·07 (11·6 to 14·59) | 0·12 | 29·53 (27·06 to 31·81) | 0·307 |
| Myanmar | 41·1 (36·66 to 45·49) | 0·284 | 54·68 (48·91 to 60·24) | 0·521 |
| Namibia | 1·41 (1·31 to 1·52) | 0·454 | 2·4 (2·11 to 2·69) | 0·612 |
| Nauru | 0·01 (0·01 to 0·01) | 0·499 | 0·01 (0·01 to 0·01) | 0·618 |
| Nepal | 19·54 (18·03 to 21·03) | 0·198 | 30·42 (26·61 to 34·24) | 0·422 |
| Netherlands | 14·92 (14·82 to 15·03) | 0·796 | 17·16 (15·68 to 18·61) | 0·883 |
| New Zealand | 3·42 (3·16 to 3·67) | 0·757 | 4·5 (4·01 to 4·97) | 0·84 |
| Nicaragua | 3·89 (3·49 to 4·26) | 0·338 | 6·51 (5·51 to 7·57) | 0·517 |
| Niger | 8·02 (7·37 to 8·64) | 0·0728 | 23·3 (20·8 to 25·93) | 0·162 |
| Nigeria | 90·19 (83·3 to 96·85) | 0·305 | 214·82 (193·13 to 236·57) | 0·515 |
| Niue | 0·002 (0·002 to 0·003) | 0·566 | 0·002 (0·001 to 0·002) | 0·711 |
| North Macedonia | 2·02 (1·84 to 2·21) | 0·618 | 2·15 (1·79 to 2·53) | 0·744 |
| Northern Mariana Islands | 0·05 (0·04 to 0·05) | 0·692 | 0·04 (0·04 to 0·05) | 0·771 |
| Norway | 4·25 (3·97 to 4·54) | 0·807 | 5·35 (4·94 to 5·75) | 0·913 |
| Oman | 1·94 (1·77 to 2·12) | 0·441 | 4·58 (4·21 to 4·95) | 0·783 |
| Pakistan | 112·84 (100·26 to 125·22) | 0·247 | 224·06 (207·08 to 241·66) | 0·449 |
| Palau | 0·02 (0·01 to 0·02) | 0·621 | 0·02 (0·02 to 0·02) | 0·738 |
| Palestine | 2·07 (1·84 to 2·31) | 0·314 | 4·96 (4·56 to 5·33) | 0·588 |
| Panama | 2·39 (2·21 to 2·55) | 0·544 | 4·16 (3·66 to 4·68) | 0·686 |
| Papua New Guinea | 4·09 (3·54 to 4·62) | 0·292 | 9·87 (8·69 to 10·95) | 0·394 |
| Paraguay | 4·05 (3·7 to 4·38) | 0·465 | 6·93 (5·7 to 8·11) | 0·638 |
| Peru | 21·73 (19·83 to 23·54) | 0·501 | 34 (31·12 to 36·63) | 0·648 |
| Philippines | 63·29 (59·12 to 67·61) | 0·497 | 112·14 (101·58 to 121·87) | 0·623 |
| Poland | 38·16 (35·13 to 41·09) | 0·632 | 38·43 (35·38 to 41·36) | 0·802 |
| Portugal | 10·14 (9·35 to 10·88) | 0·607 | 10·65 (9·43 to 11·91) | 0·743 |
| Puerto Rico | 3·61 (3·36 to 3·88) | 0·67 | 3·52 (3·11 to 3·99) | 0·814 |
| Qatar | 0·45 (0·4 to 0·49) | 0·585 | 2·86 (2·6 to 3·12) | 0·83 |
| Republic of Korea | 44·34 (41·18 to 47·17) | 0·686 | 53·4 (48·44 to 58·41) | 0·878 |
| Republic of Moldova | 4·45 (4·13 to 4·77) | 0·585 | 3·69 (3·1 to 4·33) | 0·696 |
| Romania | 23·4 (21·57 to 25·26) | 0·625 | 19·24 (17·03 to 21·54) | 0·76 |
| Russian Federation | 151·02 (139·03 to 162·58) | 0·695 | 146·72 (128·85 to 165·17) | 0·805 |
| Rwanda | 7·17 (6·65 to 7·73) | 0·257 | 12·69 (11·34 to 14·08) | 0·429 |
| Saint Kitts and Nevis | 0·04 (0·04 to 0·04) | 0·583 | 0·06 (0·05 to 0·07) | 0·746 |
| Saint Lucia | 0·14 (0·13 to 0·15) | 0·483 | 0·17 (0·15 to 0·2) | 0·67 |
| Saint Vincent and the Grenadines | 0·11 (0·1 to 0·12) | 0·462 | 0·11 (0·1 to 0·13) | 0·627 |
| Samoa | 0·16 (0·15 to 0·17) | 0·531 | 0·21 (0·19 to 0·23) | 0·641 |
| San Marino | 0·02 (0·02 to 0·03) | 0·814 | 0·03 (0·03 to 0·04) | 0·884 |
| Sao Tome and Principe | 0·12 (0·11 to 0·13) | 0·299 | 0·21 (0·18 to 0·23) | 0·502 |
| Saudi Arabia | 16·05 (14·65 to 17·36) | 0·48 | 35·73 (31·18 to 40·19) | 0·805 |
| Senegal | 7·62 (7·01 to 8·22) | 0·227 | 15·13 (13·5 to 16·85) | 0·389 |
| Serbia | 9·4 (8·63 to 10·12) | 0·626 | 8·75 (7·83 to 9·73) | 0·767 |
| Seychelles | 0·07 (0·07 to 0·08) | 0·567 | 0·1 (0·09 to 0·11) | 0·724 |
| Sierra Leone | 3·65 (3·3 to 4·02) | 0·207 | 8·28 (7·53 to 9·08) | 0·347 |
| Singapore | 3·05 (2·85 to 3·25) | 0·688 | 5·67 (5·23 to 6·06) | 0·861 |
| Slovakia | 5·28 (5·25 to 5·31) | 0·656 | 5·44 (4·97 to 5·92) | 0·812 |
| Slovenia | 1·97 (1·76 to 2·18) | 0·726 | 2·07 (1·91 to 2·24) | 0·84 |
| Solomon Islands | 0·34 (0·31 to 0·37) | 0·279 | 0·66 (0·57 to 0·74) | 0·407 |
| Somalia | 7·15 (6·55 to 7·79) | 0·0508 | 20·34 (15·2 to 25·7) | 0·081 |
| South Africa | 36·83 (32·99 to 40·74) | 0·552 | 55·59 (49·17 to 62·72) | 0·678 |
| South Sudan | 5·86 (5·18 to 6·55) | 0·248 | 9·28 (8·05 to 10·61) | 0·363 |
| Spain | 38·78 (36·19 to 41·73) | 0·647 | 46·02 (42·09 to 49·98) | 0·767 |
| Sri Lanka | 17·22 (15 to 19·31) | 0·504 | 21·85 (19·45 to 24·14) | 0·69 |
| Sudan | 20·2 (18·4 to 21·93) | 0·227 | 40·81 (35·36 to 46·01) | 0·515 |
| Suriname | 0·39 (0·34 to 0·43) | 0·498 | 0·58 (0·51 to 0·65) | 0·636 |
| Sweden | 8·59 (8·53 to 8·65) | 0·769 | 10·22 (9·31 to 11·13) | 0·872 |
| Switzerland | 6·87 (6·42 to 7·32) | 0·868 | 8·78 (8·02 to 9·56) | 0·929 |
| Syrian Arab Republic | 12·89 (11·63 to 14·09) | 0·367 | 14·49 (12·17 to 16·8) | 0·619 |
| Taiwan (Province of China) | 20·4 (20·29 to 20·52) | 0·667 | 23·62 (21·66 to 25·44) | 0·868 |
| Tajikistan | 5·38 (4·99 to 5·78) | 0·468 | 9·49 (8·21 to 10·67) | 0·539 |
| Thailand | 56·88 (53·14 to 60·82) | 0·508 | 70·11 (61·33 to 78·91) | 0·687 |
| Timor-Leste | 0·78 (0·73 to 0·84) | 0·274 | 1·33 (1·21 to 1·45) | 0·514 |
| Togo | 3·66 (3·24 to 4·09) | 0·266 | 7·92 (6·94 to 8·9) | 0·417 |
| Tokelau | 0·002 (0·002 to 0·002) | 0·427 | 0·001 (0·001 to 0·002) | 0·626 |
| Tonga | 0·1 (0·09 to 0·11) | 0·51 | 0·1 (0·09 to 0·11) | 0·636 |
| Trinidad and Tobago | 1·2 (1·12 to 1·28) | 0·618 | 1·39 (1·23 to 1·55) | 0·757 |
| Tunisia | 8·44 (7·65 to 9·24) | 0·434 | 11·57 (10·42 to 12·76) | 0·672 |
| Turkey | 59·77 (55·76 to 63·6) | 0·473 | 81·36 (71·37 to 91·24) | 0·748 |
| Turkmenistan | 3·71 (3·42 to 3·99) | 0·548 | 5·08 (4·61 to 5·54) | 0·67 |
| Tuvalu | 0·01 (0·01 to 0·01) | 0·426 | 0·01 (0·01 to 0·01) | 0·589 |
| Uganda | 17·32 (16·04 to 18·61) | 0·167 | 41·12 (37·02 to 44·96) | 0·404 |
| Ukraine | 52·66 (48·71 to 56·41) | 0·653 | 44·04 (35·75 to 52·27) | 0·736 |
| United Arab Emirates | 1·87 (1·69 to 2·06) | 0·621 | 9·24 (7·76 to 10·59) | 0·88 |
| United Kingdom | 57·47 (53·85 to 61·19) | 0·745 | 67·22 (60·47 to 73·93) | 0·847 |
| United Republic of Tanzania | 25·9 (23·78 to 28·01) | 0·26 | 56·74 (50·5 to 63·23) | 0·423 |
| United States of America | 253·61 (235·88 to 271·14) | 0·768 | 327·98 (285·96 to 369·32) | 0·859 |
| United States Virgin Islands | 0·11 (0·1 to 0·11) | 0·788 | 0·1 (0·09 to 0·12) | 0·877 |
| Uruguay | 3·14 (2·82 to 3·48) | 0·581 | 3·44 (3·03 to 3·88) | 0·697 |
| Uzbekistan | 20·95 (19·35 to 22·58) | 0·49 | 33·68 (25·41 to 42·32) | 0·631 |
| Vanuatu | 0·15 (0·14 to 0·16) | 0·361 | 0·29 (0·27 to 0·32) | 0·485 |
| Venezuela (Bolivarian Republic of) | 18·83 (17·47 to 20·19) | 0·509 | 28·07 (24·77 to 31·42) | 0·607 |
| Viet Nam | 67·94 (62·48 to 73·33) | 0·39 | 96·37 (83·07 to 109·01) | 0·617 |
| Yemen | 13·73 (12·45 to 15·06) | 0·176 | 31·5 (26·6 to 36·78) | 0·412 |
| Zambia | 7·94 (7·38 to 8·51) | 0·299 | 18·24 (15·89 to 20·47) | 0·505 |

a.Population data from GBD 2019 study, shown as central estimates (million) with 95% uncertainty intervals.

b.SDI (social-demographic index) data from GBD 2019 study. According to the GBD 2019 study, countries or territories were categorized as high SDI (0·805129, 1), high-middle SDI (0·689504, 0·805129), middle SDI (0·607679, 0·689504), low-middle SDI (0·454743, 0·607679), low SD (0, 0·454743).

Table S3. Trends of global healthy inequality in total cardiovascular diseases and five kinds of cardiovascular disease from 1990 to 2019.

| **Disease** | **Cardiovascular disease** | | **Rheumatic heart disease** | | **Ischemic heart disease** | | **Stroke** | | **Hypertensive heart disease** | | **Non-rheumatic valvular heart disease** | |
| --- | --- | --- | --- | --- | --- | --- | --- | --- | --- | --- | --- | --- |
| **Year** | **Slope index of inequality** | **Concentration index** | **Slope index of inequality** | **Concentration index** | **Slope index of inequality** | **Concentration index** | **Slope index of inequality** | **Concentration index** | **Slope index of inequality** | **Concentration index** | **Slope index of inequality** | **Concentration index** |
| 1990 | 3760.40 (3758.26 to 3756.53) | 11.75 (11.42 to 12.09) | -345.95 (-346.47 to -345.42) | -22.28 (-24.00 to -20.57) | 2833.18 (2831.67 to 2834.69) | 20.17 (19.70 to 20.65) | 788.36 (787.04 to 789.69) | 6.40 (6.13 to 6.66) | -82.07 (-82.56 to -81.59) | -5.16 (-5.60 to -4.71) | 89.79 (89.55 to 30.03) | 42.07 (40.32 to 43.82) |
| 1991 | 3729.53 (3727.41 to 3731.64) | 11.72 (11.39 to 12.05) | -345.97 (-346.49 to -345.45) | -22.65 (-24.26 to -21.03) | 2780.05 (2778.56 to 2781.55) | 19.87 (19.40 to 20.33) | 796.13 (794.82 to 797.44) | 6.51 (6.24 to 6.78) | -80.76 (-81.23 to -80.28) | -5.08 (-5.50 to -4.67) | 90.72 (90.48 to 90.96) | 42.27 (40.53 to 44.02) |
| 1992 | 3775.09 (3772.99 to 3777.18) | 11.85 (11.53 to 12.17) | -344.74 (-345.25 to -344.23) | -22.92 (-24.60 to -21.24) | 2780.33 (2778.85 to 2781.82) | 19.80 (19.35 to 20.26) | 822.12 (820.82 to 823.42) | 6.71 (6.44 to 6.99) | -77.09 (-77.56 to -76.62) | -4.89 (-5.27 to -4.50) | 91.95 (91.71 to 92.19) | 42.50 (40.79 to 44.20) |
| 1993 | 4124.28 (4122.18 to 4126.39) | 12.79 (12.48 to 13.11) | -331.65 (-332.15 to -331.15) | -22.55 (-24.11 to -20.99) | 2968.28 (2966.78 to 2969.78) | 20.71 (20.26 to 21.15) | 938.50 (937.20 to 939.80) | 7.59 (7.31 to 7.87) | -69.85 (-70.32 to -69.39) | -4.48 (-4.81 to -4.16) | 93.95 (93.71 to 94.19) | 42.87 (41.21 to 44.53) |
| 1994 | 4251.65 (4249.56 to 4253.74) | 13.15 (12.84 to 13.45) | -324.48 (-324.97 to -323.99) | -22.52 (-24.03 to -21.01) | 3007.74 (3006.24 to 3009.23) | 20.82 (20.41 to 21.23) | 997.37 (996.08 to 998.67) | 8.05 (7.77 to 8.34) | -63.37 (-63.83 to -62.91) | -4.13 (-4.40 to -3.86) | 93.97 (93.73 to 94.21) | 42.83 (41.20 to 44.47) |
| 1995 | 4192.37 (4190.30 to 4194.44) | 13.08 (12.78 to 13.37) | -322.96 (-323.44 to -322.48) | -23.02 (-24.56 to -21.49) | 2927.94 (2926.47 to 2929.41) | 20.48 (20.07 to 20.88) | 998.25 (996.97 to 999.54) | 8.11 (7.82 to 8.39) | -59.21 (-59.66 to -58.76) | -3.91 (-4.15 to -3.68) | 94.05 (93.81 to 94.28) | 42.87 (41.28 to 44.46) |
| 1996 | 3922.28 (3920.24 to 3924.32) | 12.37 (12.10 to 12.64) | -329.51 (-329.98 to -329.03) | -24.00 (-25.58 to -22.41) | 2719.10 (2717.66 to 2720.54) | 19.30 (18.93 to 19.68) | 938.92 (937.65 to 940.19) | 7.68 (7.43 to 7.93) | -57.03 (-57.47 to -56.58) | -3.80 (-4.03 to -3.57) | 92.72 (92.49 to 92.95) | 42.58 (40.99 to 44.16) |
| 1997 | 3596.16 (3594.15 to 3598.17) | 11.42 (11.18 to 11.67) | -341.49 (-341.96 to -341.01) | -25.13 (-26.84 to -23.43) | 2482.22 (2480.81 to 2483.63) | 17.80 (17.45 to 18.15) | 861.10 (859.85 to 862.36) | 7.07 (6.86 to 7.29) | -55.43 (-55.87 to -54.99) | -3.72 (-3.93 to -3.51) | 91.18 (90.95 to 91.41) | 42.14 (40.49 to 43.79) |
| 1998 | 3491.70 (3489.71 to 3493.68) | 11.17 (10.93 to 11.41) | -345.51 (-345.99 to -345.04) | -25.81 (-27.60 to -24.02) | 2383.15 (2381.76 to 2384.54) | 17.26 (16.93 to 17.59) | 847.21 (845.97 to 848.76) | 6.99 (6.78 to 7.21) | -50.46 (-50.90 to -50.03) | -3.41 (-3.60 to -3.23) | 90.78 (90.55 to 91.01) | 42.07 (40.39 to 43.74) |
| 1999 | 3656.71 (3654.73 to 3658.69) | 11.65 (11.40 to 11.90) | -343.40 (-343.87 to -342.93) | -25.99 (-27.73 to -24.24) | 2444.86 (2443.47 to 2446.25) | 17.62 (17.28 to 17.96) | 921.22 (919.98 to 922.47) | 7.56 (7.33 to 7.80) | -40.91 (-41.34 to -40.47) | -2.77 (-2.91 to -2.63) | 89.45 (89.23 to 89.68) | 41.82 (40.16 to 43.47) |
| 2000 | 3728.07 (3726.10 to 3730.04) | 11.85 (11.59 to 12.11) | -329.69 (-330.14 to -329.23) | -25.52 (-27.23 to -23.80) | 2422.84 (2421.46 to 2424.22) | 17.43 (17.10 to 17.77) | 971.10 (969.86 to 972.34) | 7.92 (7.68 to 8.16) | -28.74 (-29.17 to -28.31) | -1.95 (-2.05 to -1.86) | 87.98 (87.76 to 88.20) | 41.53 (39.85 to 43.20) |
| 2001 | 3675.92 (3673.96 to 3677.88) | 11.68 (11.42 to 11.93) | -323.09 (-323.54 to -322.64) | -25.72 (-27.45 to -24.00) | 2329.48 (2328.11 to 2330.84) | 16.73 (16.41 to 17.06) | 978.19 (976.97 to 979.42) | 7.98 (7.74 to 8.22) | -18.09 (-18.52 to -17.67) | -1.24 (-1.30 to -1.18) | 87.38 (87.16 to 87.60) | 41.45 (39.77 to 43.12) |
| 2002 | 3845.23 (3843.28 to 3847.19) | 12.14 (11.87 to 12.41) | -291.15 (-291.58 to -290.72) | -24.06 (-25.63 to -22.48) | 2286.10 (2284.74 to 2287.46) | 16.26 (15.94 to 16.58) | 1129.71 (1128.48 to 1130.94) | 9.17 (8.90 to 9.45) | -17.16 (-17.58 to -16.74) | -1.18 (-1.24 to -1.13) | 88.38 (88.16 to 88.60) | 41.71 (40.09 to 43.33) |
| 2003 | 4009.55 (4007.60 to 4011.50) | 12.66 (12.40 to 12.93) | -275.15 (-275.57 to -274.74) | -23.49 (-25.00 to -21.98) | 2317.21 (2315.86 to 2318.57) | 16.43 (16.11 to 16.75) | 1290.73 (1289.50 to 1291.95) | 10.51 (10.21 to 10.08) | -7.35 (-7.77 to -6.94) | -0.51 (-0.54 to -0.48) | 86.86 (86.65 to 87.08) | 41.15 (39.58 to 42.72) |
| 2004 | 3956.14 (3954.21 to 3958.06) | 12.64 (12.37 to 12.90) | -265.34 (-265.75 to -264.93) | -23.30 (-24.76 to -21.84) | 2252.40 (2251.07 to 2253.74) | 16.17 (15.85 to 16.48) | 1228.58 (1227.37 to 1229.79) | 10.13 (9.85 to 10.41) | -0.50 (-0.91 to -0.09) | -0.04 (-0.06 to -0.01) | 85.80 (85.58 to 86.01) | 40.93 (39.44 to 42.43) |
| 2005 | 4015.98 (4014.06 to 4017.90) | 12.80 (12.52 to 13.08) | -265.99 (-266.40 to -265.59) | -23.68 (-25.19 to -22.16) | 2244.55 (2243.22 to 2245.88) | 16.01 (15.68 to 16.34) | 1213.11 (1211.91 to 1214.31) | 10.07 (9.78 to 10.35) | 7.35 (6.94 to 7.75) | 0.52 (0.47 to 0.57) | 87.06 (86.85 to 87.27) | 41.17 (39.69 to 42.66) |
| 2006 | 3684.64 (3682.76 to 3686.53) | 11.93 (11.68 to 12.18) | -267.00 (-267.40 to -266.60) | -24.30 (-25.95 to -22.66) | 2016.05 (2014.75 to 2017.36) | 14.56 (14.27 to 14.86) | 1164.75 (1163.57 to 1165.3) | 9.90 (9.63 to 10.16) | 13.63 (13.23 to 14.04) | 0.96 (0.89 to 1.03) | 87.31 (87.10 to 87.53) | 41.12 (39.63 to 42.62) |
| 2007 | 3439.92 (3438.06 to 3441.78) | 11.22 (10.98 to 11.46) | -268.07 (-268.47 to -267.68) | -24.91 (-26.54 to -23.27) | 1875.67 (1874.38 to 1876.95) | 13.59 (13.31 to 13.88) | 1037.74 (1036.58 to 1038.90) | 8.95 (8.71 to 9.19) | 20.61 (20.21 to 21.02) | 1.44 (1.35 to 1.53) | 88.69 (88.47 to 88.90) | 41.31 (39.80 to 42.80) |
| 2008 | 3377.86 (3376.01 to 3379.71) | 11.00 (10.76 to 11.23) | -270.20 (-270.60 to -269.81) | -25.40 (-27.04 to -23.76) | 1842.54 (1841.27 to 1843.82) | 13.27 (12.98 to 13.55) | 1030.07 (1028.92 to 1031.22) | 8.92 (8.68 to 9.16) | 33.00 (32.60 to 33.40) | 2.27 (2.14 to 2.40) | 89.53 (89.32 to 89.75) | 41.25 (39.75 to 42.75) |
| 2009 | 3377.31 (3375.48 to 3379.15) | 11.07 (10.84 to 11.30) | -261.29 (-261.67 to -260.90) | -25.20 (-26.81 to -23.58) | 1733.43 (1732.16 to 1734.69) | 12.55 (12.29 to 12.82) | 1101.05 (1099.91 to 1102.19) | 9.61 (9.36 to 9.87) | 53.00 (52.59 to 53.40) | 3.60 (3.40 to 3.80) | 90.77 (90.56 to 90.99) | 41.43 (39.94 to 42.92) |
| 2010 | 3352.78 (3350.95 to 3354.60) | 10.97 (10.71 to 11.23) | -257.36 (-257.74 to -256.98) | -25.23 (-26.90 to -23.57) | 1694.54 (1693.28 to 1695.79) | 12.19 (11.91 to 12.48) | 1108.86 (1107.73 to 1109.99) | 9.71 (9.43 to 10.00) | 64.83 (64.43 to 65.24) | 4.33 (4.09 to 4.57) | 92.25 (92.04 to 92.47) | 41.58 (40.10 to 43.05) |
| 2011 | 3216.91 (3215.11 to 3218.72) | 10.57 (10.31 to 10.82) | -248.61 (-248.98 to -248.23) | -24.89 (-26.53 to -23.24) | 1578.65 (1577.41 to 1579.90) | 11.35 (11.07 to 11.63) | 1128.33 (1127.21 to 1129.45) | 9.97 (9.67 to 10.27) | 71.40 (70.99 to 71.81) | 4.68 (4.40 to 4.97) | 92.81 (92.60 to 93.03) | 41.29 (39.81 to 42.77) |
| 2012 | 3120.70 (3118.91 to 3122.49) | 10.28 (10.03 to 10.53) | -242.01 (-242.38 to -241.65) | -24.89 (-26.62 to -23.16) | 1489.94 (1488.71 to 1491.18) | 10.70 (10.45 to 10.96) | 1098.75 (1097.64 to 1099.86) | 9.79 (9.50 to 10.09) | 77.65 (77.24 to 78.06) | 4.99 (4.68 to 5.31) | 94.79 (94.58 to 95.01) | 41.57 (40.07 to 43.07) |
| 2013 | 3071.69 (3069.91 to 3073.46) | 10.15 (9.90 to 10.40) | -238.28 (-238.64 to -237.92) | -25.01 (-26.82 to -23.21) | 1465.79 (1464.56 to 1467.01) | 10.54 (10.28 to 10.80) | 1070.78 (1069.68 to 1071.88) | 9.63 (9.33 to 9.92) | 85.29 (84.88 to 85.71) | 5.37 (5.01 to 5.74) | 95.58 (95.36 to 95.79) | 41.42 (39.92 to 42.92) |
| 2014 | 3115.70 (3113.94 to 3117.47) | 10.33 (10.06 to 10.60) | -229.88 (-230.24 to -229.53) | -24.76 (-26.52 to -22.99) | 1482.48 (1481.26 to 1483.70) | 10.70 (10.43 to 10.97) | 1086.44 (1085.35 to 1087.53) | 9.82 (9.50 to 10.14) | 94.89 (94.48 to 95.31) | 5..86 (5.44 to 6.29) | 96.78 (96.57 to 97.00) | 41.49 (40.00 to 42.98) |
| 2015 | 3220.28 (3218.51 to 3222.04) | 10.59 (10.29 to 10.88) | -225.92 (-226.27 to -225.57) | -24.65 (-26.48 to -22.81) | 1527.08 (1525.85 to 1528.30) | 10.87 (10.58 to 11.17) | 1118.61 (1117.52 to 1119.69) | 10.08 (9.73 to 10.43) | 102.49 (102.08 to 102.91) | 6.23 (5.75 to 6.71) | 98.56 (98.35 to 98.77) | 41.51 (40.03 to 42.99) |
| 2016 | 3212.03 (3210.28 to 3213.79) | 10.53 (10.21 to 10.85) | -220.98 (-221.32 to -220.64) | -24.48 (-26.32 to -22.63) | 1498.86 (1497.65 to 1500.07) | 10.64 (10.32 to 10.97) | 1127.94 (1126.85 to 1129.02) | 10.13 (9.74 to 10.51) | 107.12 (106.70 to 107.53) | 6.44 (5.93 to 6.94) | 99.18 (98.96 to 99.39) | 41.38 (39.87 to 42.88) |
| 2017 | 3194.48 (3192.73 to 3196.22) | 10.46 (10.11 to 10.81) | -218.19 (-218.53 to -217.85) | -24.48 (-26.36 to -22.60) | 1474.91 (1473.70 to 1476.12) | 10.46 (10.11 to 10.81) | 1132.04 (1130.96 to 1133.11) | 10.15 (9.74 to 10.57) | 107.87 (107.46 to 108.29) | 6.46 (5.95 to 6.97) | 98.98 (98.77 to 99.19) | 41.16 (39.61 to 42.70) |
| 2018 | 3290.33 (3288.59 to 3292.08) | 10.69 (10.29 to 11.10) | -212.31 (-212.64 to -211.98) | -24.09 (-26.19 to -22.00) | 1522.17 (1520.96 to 1523.38) | 10.69 (10.26 to 11.12) | 1179.07 (1178.00 to 1180.15) | 10.51 (10.04 to 10.98) | 105.01 (104.59 to 105.42) | 6.27 (5.76 to 6.77) | 99.66 (99.45 to 99.88) | 40.95 (39.37 to 42.53) |
| 2019 | 3400.38 (3398.64 to 3402.13) | 10.96 (10.52 to 11.40) | -204.34 (-204.67 to -204.01) | -23.55 (-25.80 to -21.30) | 1560.28 (1559.07 to 1561.48) | 10.85 (10.39 to 11.32) | 1237.48 (1236.40 to 1238.55) | 10.94 (10.41 to 11.46) | 108.99 (108.57 to 109.40) | 6.45 (5.95 to 6.95) | 100.95 (100.74 to 101.16) | 40.96 (39.30 to 42.62) |

Central estimates with 95% confidence intervals.

Table S4. Trends of global healthy inequality in five other types of cardiovascular disease from 1990 to 2019.

| **Disease** | **Cardiomyopathy and myocarditis** | | **Atrial fibrillation and flutter** | | **Aortic aneurysm** | | **Peripheral artery disease** | | **Endocarditis** | |
| --- | --- | --- | --- | --- | --- | --- | --- | --- | --- | --- |
| **Year** | **Slope index of inequality** | **Concentration index** | **Slope index of inequality** | **Concentration index** | **Slope index of inequality** | **Concentration index** | **Slope index of inequality** | **Concentration index** | **Slope index of inequality** | **Concentration index** |
| 1990 | 273.05 (272.62 to 273.47) | 31.90 (29.22 to 34.58) | 163.01 (162.69 to 163.34) | 35.02 (32.62 to 37.42) | 104.91 (104.65 to 105.17) | 41.36 (39.48 to 43.25) | 55.46 (55.27 to 55.66) | 51.81 (44.71 to 58.91) | -4.50 (-4.64 to -4.36) | -3.53 (-3.86 to -3.19) |
| 1991 | 278.12 (277.69 to 278.54) | 32.42 (29.77 to 35.07) | 162.08 (161.76 to 162.41) | 34.76 (32.43 to 37.09 | 107.65 (107.39 to 107.91) | 41.71 (39.83 to 43.58) | 55.57 (55.37 to 55.76) | 51.66 (44.53 to 58.80) | -3.78 (-3.91 to -3.64) | -2.96 (-3.23 to -2.68) |
| 1992 | 289.32 (288.89 to 289.75) | 33.23 (30.56 to 35.90) | 161.69 (161.37 to 162.01) | 34.58 (32.29 to 36.86) | 110.11 (109.85 to 110.38) | 42.01 (40.20 to 43.83) | 55.93 (55.74 to 56.13) | 51.58 (44.44 to 58.73) | -2.82 (-2.96 to -2.69) | -2.21 (-2.40 to -2.01) |
| 1993 | 309.87 (309.43 to 310.31) | 34.50 (31.60 to 37.40) | 162.73 (162.41 to 163.05) | 34.50 (32.26 to 36.74) | 113.46 (113.19 to 113.72) | 42.32 (40.58 to 44.06) | 56.63 (56.44 to 56.83) | 51.59 (44.39 to 58.79) | -1.63 (-1.76 to --1.49) | -1.26 (-1.38 to --1.15) |
| 1994 | 323.39 (322.95 to 323.83) | 35.23 (32.15 to 38.32) | 162.92 (162.60 to 163.24) | 34.37 (32.13 to 36.61) | 114.46 (114.20 to 114.73) | 42.23 (40.54 to 43.92) | 56.85 (56.66 to 57.04) | 51.41 (44.18 to 58.65) | -0.22 (-0.36 to -0.09) | -0.17 (-0.21 to -0.13) |
| 1995 | 327.57 (323.12 to 324.01) | 35.39 (32.60 to 38.17) | 164.05 (163.74 to 164.37) | 34.36 (32.11 to 36.62) | 115.09 (114.83 to 115.35) | 42.23 (40.61 to 43.86) | 57.08 (56.89 to 57.27) | 51.33 (44.14 to 58.52) | 1.15 (1.01 to 1.28) | 0.87 (0.78 to 0.96) |
| 1996 | 317.00 (316.57 to 317.43) | 35.28 (32.88 to 37.67) | 164.75 (164.43 to 165.06) | 34.27 (32.03 to 36.51) | 113.83 (113.57 to 114.09) | 41.87 (40.28 to 43.47) | 57.21 (57.02 to 57.40) | 51.25 (44.13 to 58.38) | 2.27 (2.13 to 2.40) | 1.71 (1.54 to 1.87) |
| 1997 | 307.30 (306.87 to 307.73) | 34.91 (32.72 to 37.10) | 165.14 (164.83 to 165.45) | 34.11 (31.88 to 36.34) | 111.84 (111.58 to 112.09) | 41.29 (39.69 to 42.89) | 57.19 (57.00 to 57.37) | 51.09 (44.01 to 58.17) | 3.45 (3.32 to 3.58) | 2.60 (2.36 to 2.83) |
| 1998 | 305.28 (304.86 to 305.70) | 34.93 (32.86 to 37.01) | 166.81 (166.50 to 167.12) | 34.14 (31.90 to 36.37) | 111.95 (111.70 to 112.20) | 41.26 (39.69 to 42.84) | 57.48 (57.30 to 57.67) | 51.09 (44.03 to 58.16) | 5.63 (5.50 to 5.76) | 4.20 (3.84 to 4.56) |
| 1999 | 315.13 (314.70 to 315.55) | 35.56 (33.55 to 37.57) | 169.47 (169.16 to 169.78) | 34.25 (32.02 to 36.47) | 113.61 (113.36 to 113.86) | 41.52 (40.03 to 43.01) | 58.24 (58.06 to 58.43) | 51.20 (44.04 to 58.35) | 8.94 (8.81 to 9.07) | 6.54 (6.02 to 7.05) |
| 2000 | 331.31 (330.88 to 331.74) | 36.46 (34.61 to 38.32) | 171.89 (171.57 to 172.20) | 34.33 (32.08 to 36.58) | 111.97 (111.72 to 112.22) | 41.09 (39.62 to 42.56) | 58.54 (58.36 to 58.73) | 51.12 (43.99 to 58.25) | 11.60 (11.47 to 11.74) | 8.42 (7.81 to 9.03) |
| 2001 | 344.70 (344.27 to 345.14) | 37.26 (35.53 to 38.99) | 174.82 (174.51 to 175.13) | 34.43 (32.17 to 36.68) | 110.08 (109.83 to 110.32) | 40.77 (39.31 to 42.24) | 58.93 (58.775 to 59.12) | 51.14 (44.03 to 58.25) | 13.87 (13.73 to 14.00) | 10.11 (9.45 to 10.78) |
| 2002 | 367.42 (366.97 to 667.87) | 38.29 (36.57 to 40.01) | 181.60 (181.28 to 181.91) | 34.98 (32.67 to 37.28) | 109.95 (109.70 to 110.19) | 40.67 (39.25 to 42.09) | 59.94 (59.75 to 60.13) | 51.30 (44.14 to 58.45) | 13.51 (13.38 to 13.64) | 9.91 (9.30 to 10.52) |
| 2003 | 368.14 (367.69 to 368.58) | 37.38 (35.73 to 39.04) | 185.08 (184.76 to 185.40) | 35.02 (32.75 to 37.29) | 102.97 (102.73 to 103.20) | 38.67 (37.34 to 40.00) | 58.98 (58.79 to 59.16) | 50.42 (43.37 to 57.46) | 13.09 (12.96 to 13.22) | 9.66 (9.10 to 10.23) |
| 2004 | 387.19 (386.73 to 387.67) | 38.17 (36.38 to 39.97) | 187.60 (187.28 to 187.91) | 35.03 (32.74 to 37.33) | 100.39 (100.16 to 100.62) | 38.23 (37.00 to 39.45) | 59.27 (59.09 to 59.45) | 50.35 (43.29 to 57.41) | 13.53 (13.41 to 13.66) | 10.08 (9.49 to 10.67) |
| 2005 | 430.83 (430.36 to 431.31) | 39.92 (37.42 to 42.43) | 191.76 (191.44 to 192.08) | 35.14 (32.86 to 37.42) | 99.51 (99.28 to 99.74) | 37.92 (36.67 to 39.18) | 59.70 (59.52 to 59.89) | 50.21 (43.07 to 57.35) | 13.51 (13.38 to 13.64) | 10.02 (9.45 to 10.59) |
| 2006 | 417.63 (417.17 to 418.10) | 39.58 (37.07 to 42.07) | 194.65 (194.33 to 194.97) | 35.12 (32.80 to 37.43) | 96.29 (96.07 to 96.52) | 37.12 (35.89 to 38.35) | 60.20 (60.02 to 60.38) | 50.25 (43.14 to 57.36) | 14.03 (13.90 to 14.16) | 10.46 (9.85 to 11.07) |
| 2007 | 402.01 (401.56 to 402.46) | 39.09 (36.48 to 41.70) | 198.86 (198.54 to 199.18) | 35.19 (32.86 to 37.52) | 94.10 (93.88 to 94.32) | 36.49 (35.23 to 37.72) | 60.52 (60.34 to 60.70) | 50.14 (43.05 to 57.24) | 14.38 (14.25 to 14.51) | 10.72 (10.10 to 11.33) |
| 2008 | 391.72 (391.28 to 392.17) | 38.73 (36.14 to 41.32) | 202.74 (202.42 to 203.06) | 35.14 (32.81 to 37.47) | 91.97 (91.76 to 92.19) | 35.69 (34.45 to 36.92) | 60.95 (60.77 to 61.13) | 49.93 (42.82 to 57.03) | 14.56 (14.52 to 14.78) | 10.89 (10.26 to 11.52) |
| 2009 | 363.57 (363.14 to 354.00) | 37.72 (35.24 to 40.20) | 208.08 (207.76 to 208.40) | 35.37 (33.00 to 37.75) | 90.16 (89.95 to 90.37) | 35.20 (34.02 to 36.39) | 61.28 (61.10 to 61.46) | 49.81 (42.72 to 56.89) | 14.54 (14.42 to 14.67) | 10.85 (10.23 to 11.47) |
| 2010 | 344.57 (344.16 to 344.99) | 36.98 (34.63 to 39.34) | 211.90 (211.58 to 212.23) | 35.35 (32.96 to 37.74) | 88.99 (88.78 to 89.20) | 34.69 (33.52 to 35.87) | 61.70 (61.52 to 61.88) | 49.52 (42.48 to 56.56) | 14.78 (14.65 to 14.90) | 11.01 (10.39 to 11.63) |
| 2011 | 307.93 (307.54 to 308.32) | 35.27 (33.18 to 37.35) | 216.49 (216.17 to 216.82) | 35.42 (33.03 to 37.81) | 87.65 (87.44 to 87.86) | 34.22 (33.04 to 35.41) | 62.25 (62.07 to 62.43) | 49.38 (42.33 to 56.42) | 14.52 (14.39 to 14.64) | 10.81 (10.20 to 11.43) |
| 2012 | 290.45 (290.07 to 290.83) | 34.47 (32.50 to 36.43) | 220.35 (220.03 to 220.68) | 35.37 (32.94 to 37.80) | 87.24 (87.03 to 87.45) | 34.01 (32.77 to 35.24) | 63.06 (62.88 to 63.24) | 49.28 (42.20 to 56.36) | 14.89 (14.77 to 15.01) | 11.08 (10.45 to 11.72) |
| 2013 | 276.22 (275.86 to 276.59) | 33.66 (31.78 to 35.55) | 223.04 (222.71 to 223.36) | 35.13 (32.67 to 37.60) | 86.39 (86.19 to 86.60) | 33.62 (32.43 to 34.80) | 63.54 (63.37 to 63.72) | 49.01 (42.01 to 56.02) | 14.90 (14.78 to 15.03) | 11.07 (10.45 to 11.69) |
| 2014 | 270.16 (269.80 to 270.52) | 33.43 (31.62 to 35.25) | 225.63 (225.30 to 225.96) | 34.90 (32.41 to 37.40) | 86.40 (86.20 to 86.60) | 33.40 (32.24 to 34.56) | 64.22 (64.04 to 64.40) | 48.81 (41.83 to 55.79) | 15.53 (15.41 to 15.65) | 11.50 (10.85 to 12.15) |
| 2015 | 270.48 (270.13 to 270.84) | 33.69 (31.87 to 35.51) | 230.22 (229.89 to 230.55) | 34.87 (32.44 to 37.30) | 87.45 (87.25 to 87.66) | 33.31 (32.19 to 34.43) | 65.17 (64.99 to 65.34) | 48.71 (41.72 to 55.69) | 16.14 (16.02 to 16.26) | 11.84 (11.17 to 12.51) |
| 2016 | 264.11 (263.76 to 264.47) | 33.46 (31.68 to 35.23) | 233.19 (232.86 to 233.52) | 34.70 (32.24 to 37.16) | 87.96 (87.76 to 88.17) | 33.05 (31.90 to 34.21) | 65.80 (65.62 to 65.98) | 48.52 (41.54 to 55.49) | 16.26 (16.14 to 16.38) | 11.90 (11.22 to 12.58) |
| 2017 | 257.18 (256.84 to 257.53) | 33.19 (31.42 to 34.96) | 235.67 (235.34 to 236.00) | 34.48 (31.98 to 36.98) | 88.60 (88.40 to 88.81) | 32.90 (31.71 to 34.09) | 66.07 (65.89 to 66.24) | 48.20 (41.24 to 55.15) | 16.20 (16.08 to 16.32) | 11.95 (11.25 to 12.65) |
| 2018 | 253.38 (253.04 to 253.72) | 32.83 (30.86 to 34.80) | 240.96 (240.63 to 241.29) | 34.48 (31.96 to 36.99) | 89.62 (89.41 to 89.82) | 32.66 (31.35 to 33.97) | 66.54 (66.36 to 66.72) | 47.70 (40.73 to 54.66) | 15.89 (15.77 to 16.01) | 11.68 (10.98 to 12.37) |
| 2019 | 250.76 (250.42 to 251.09) | 32.59 (30.49 to 34.69) | 245.43 (245.09 to 245.76) | 34.42 (31.97 to 36.87) | 91.14 (90.94 to 91.35) | 32.57 (31.25 to 33.89) | 67.87 (67.70 to 68.05) | 47.67 (40.72 to 54.61) | 16.00 (15.88 to 16.12) | 11.72 (11.02 to 12.43) |

Central estimates with 95% confidence intervals.


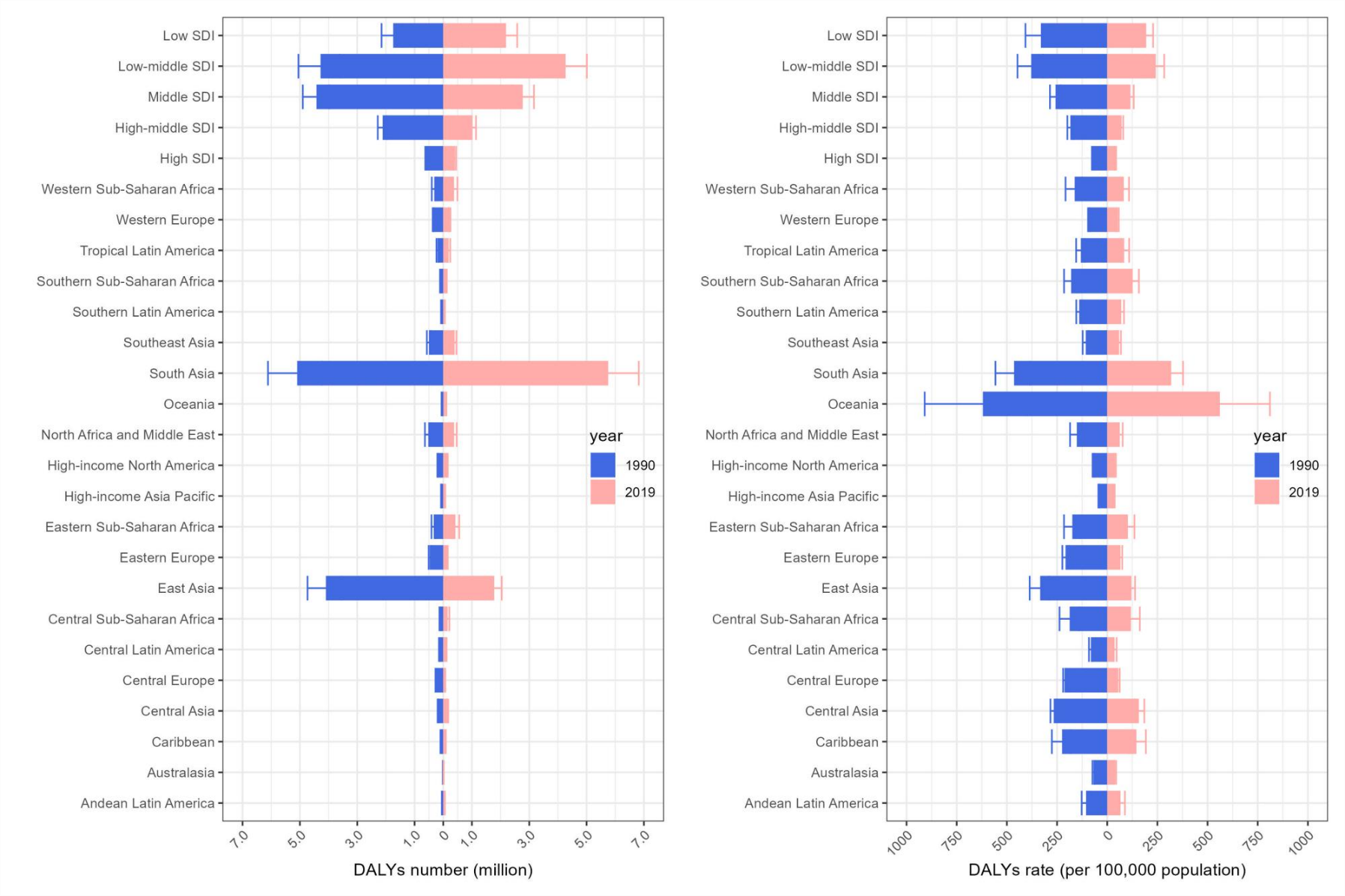
Figure S1. Change in burden of rheumatic heart disease by region, 1990 vs 2019. Columns and error bar representing the central estimates and 95% uncertainty interval of DALYs cases (A)and DALYs rates (B) in all ages population, respectively. DALYs, disability-adjusted life-years; SDI, Sociodemographic Index.

Figure S2. Change in burden of ischemic heart disease by region, 1990 vs 2019. Columns and error bar representing the central estimates and 95% uncertainty interval of DALYs cases


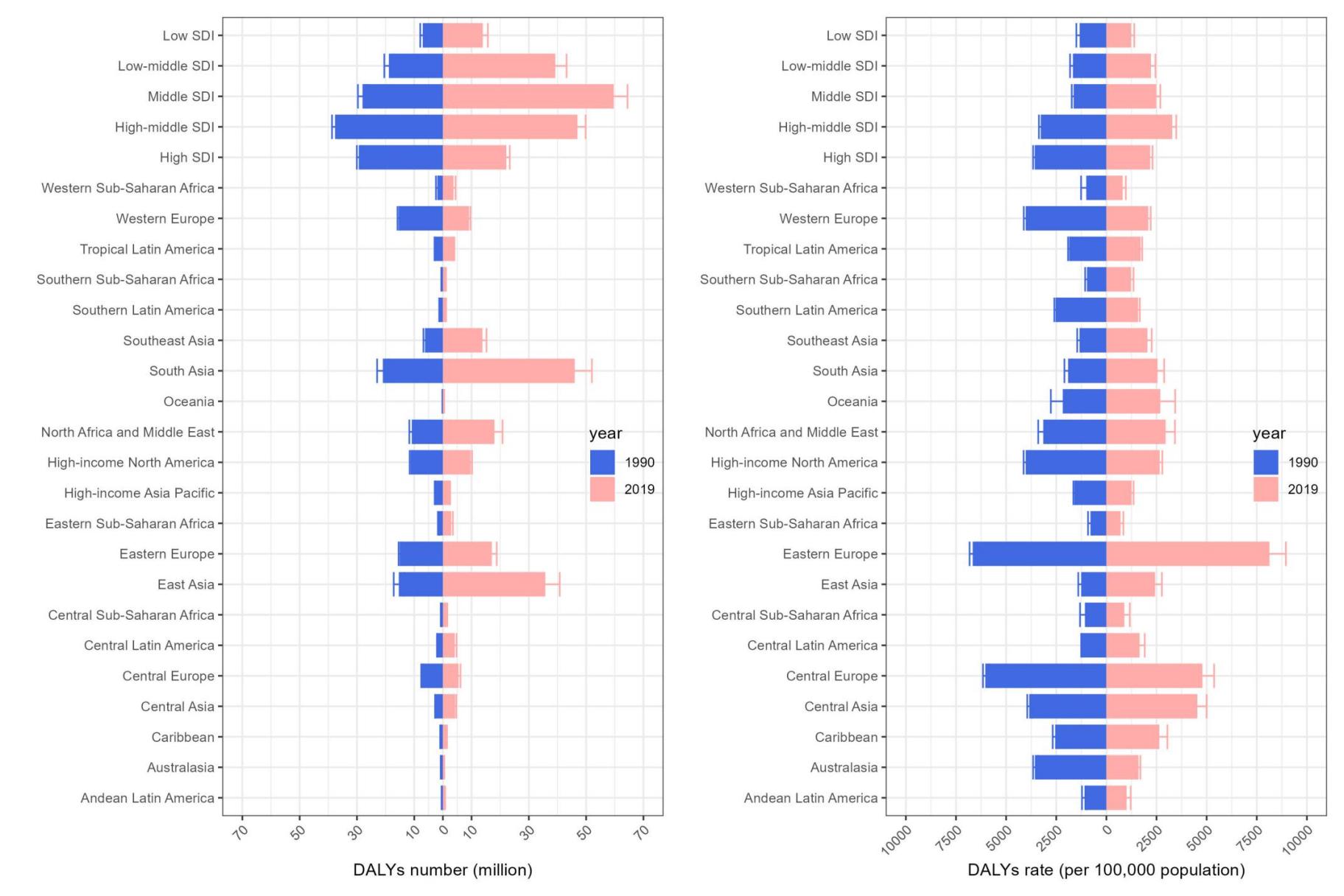
(A)and DALYs rates (B) in all ages population, respectively. DALYs, disability-adjusted life-years; SDI, Sociodemographic Index.

Figure S3. Change in burden of stroke by region, 1990 vs 2019. Columns and error bar representing the central estimates and 95% uncertainty interval of DALYs cases


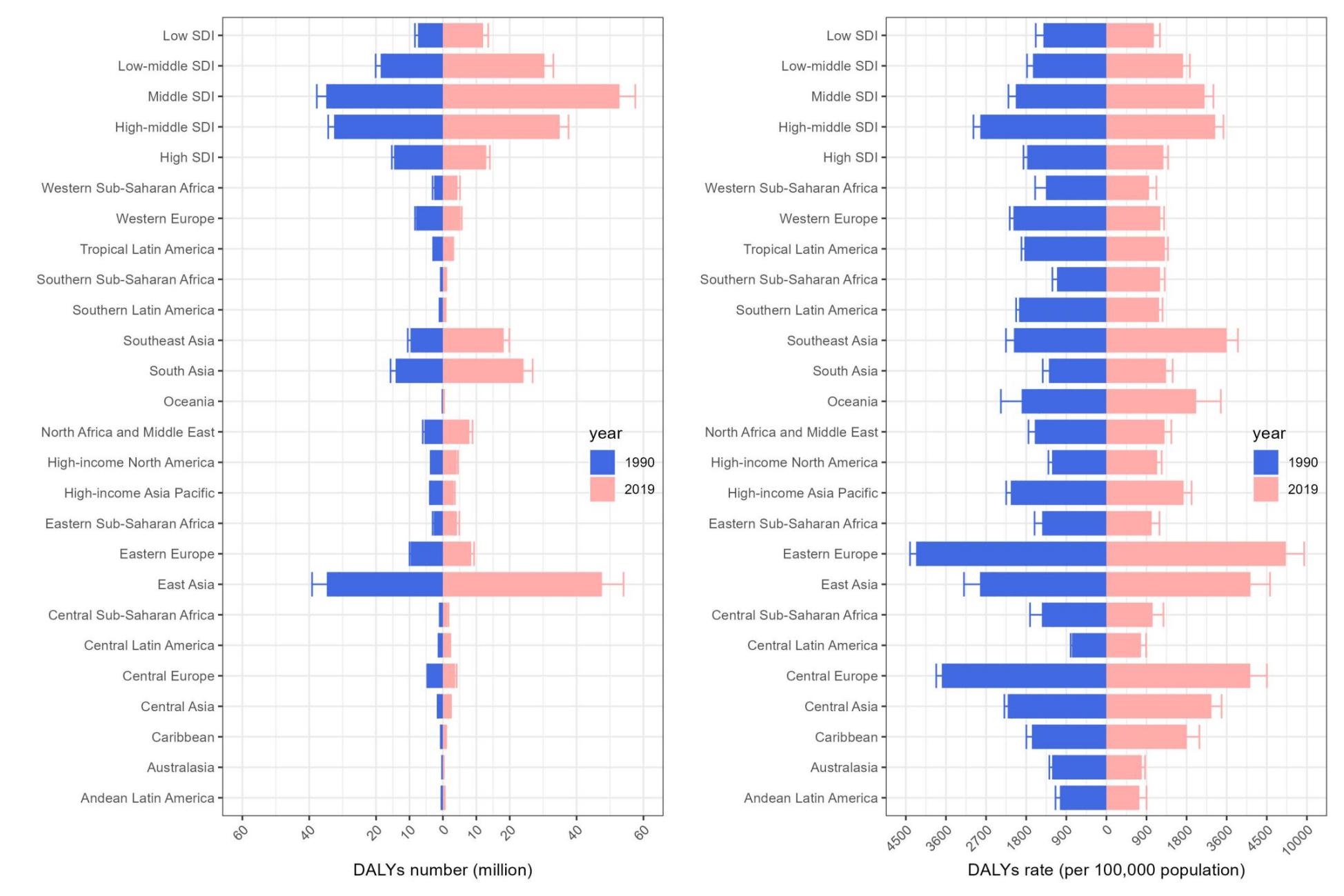
(A) and DALYs rates (B) in all ages population, respectively. DALYs, disability-adjusted life-years; SDI, Sociodemographic Index.


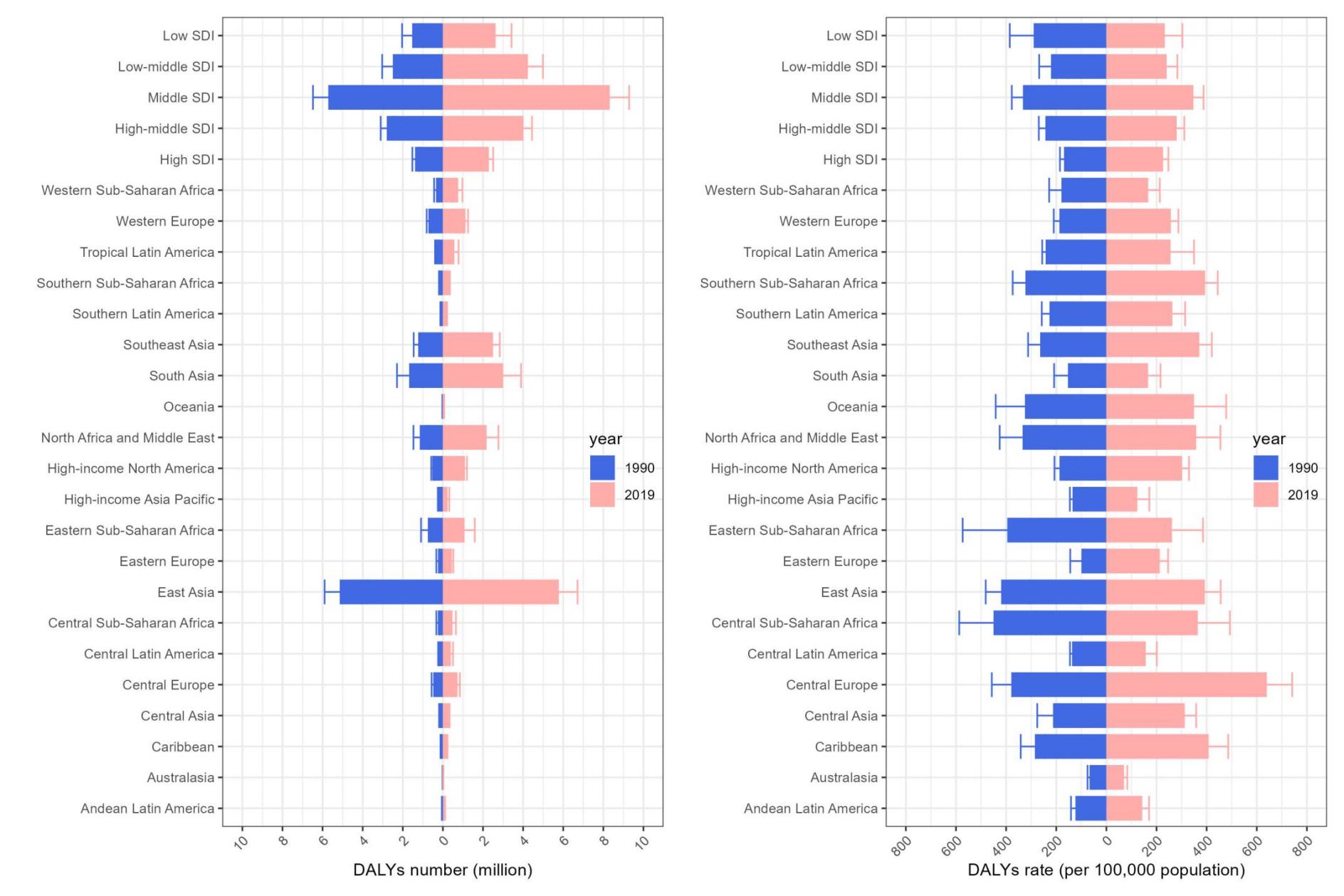
Figure S4. Change in burden of hypertensive heart disease by region, 1990 vs 2019. Columns and error bar representing the central estimates and 95% uncertainty interval of DALYs cases (A) and DALYs rates (B) in all ages population, respectively. DALYs, disability-adjusted life-years; SDI, Sociodemographic Index.


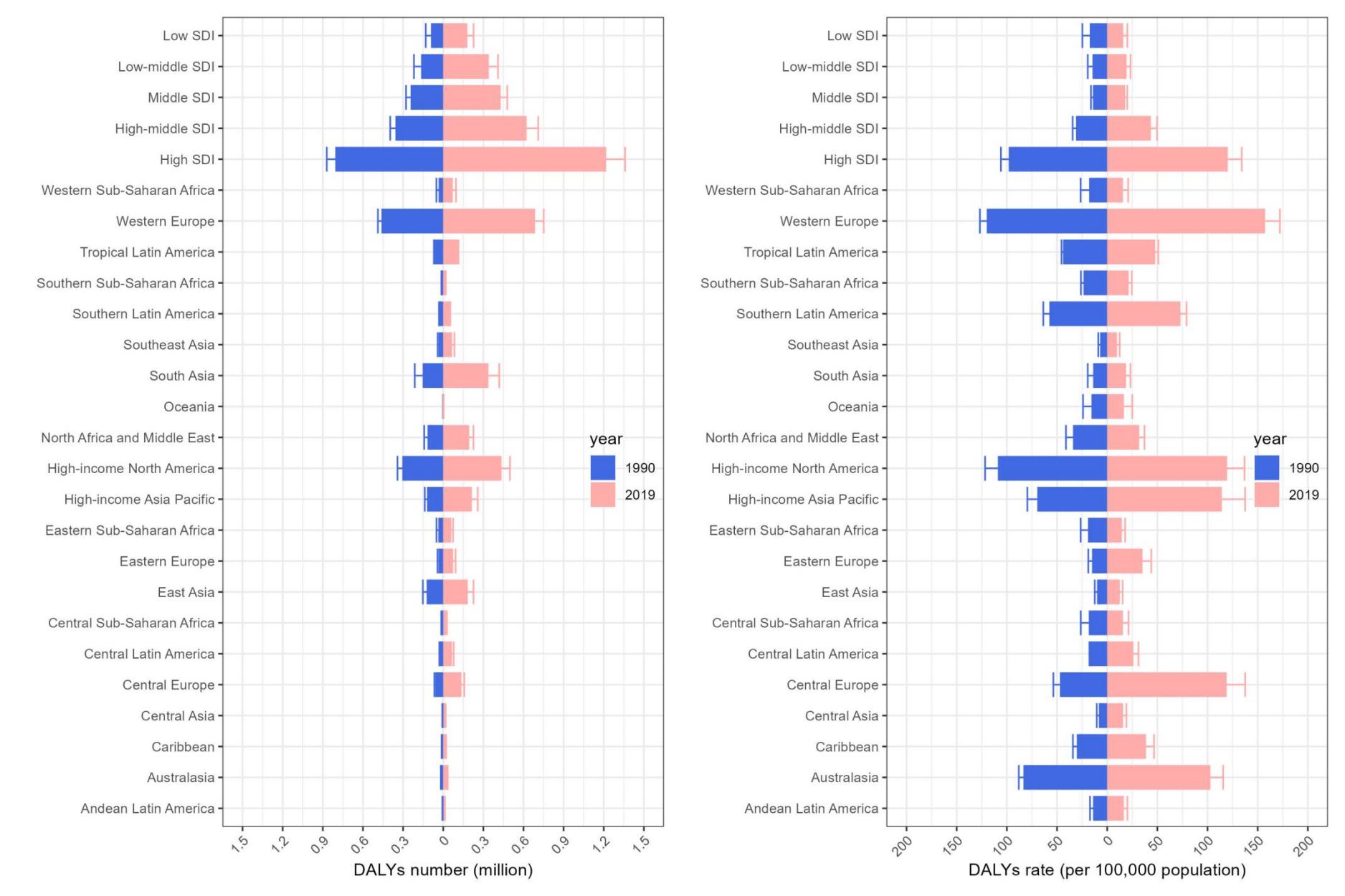
Figure S5. Change in burden of non-rheumatic valvular heart disease by region, 1990 vs 2019. Columns and error bar representing the central estimates and 95% uncertainty interval of DALYs cases (A) and DALYs rates (B) in all ages population, respectively. DALYs, disability-adjusted life-years; SDI, Sociodemographic Index.


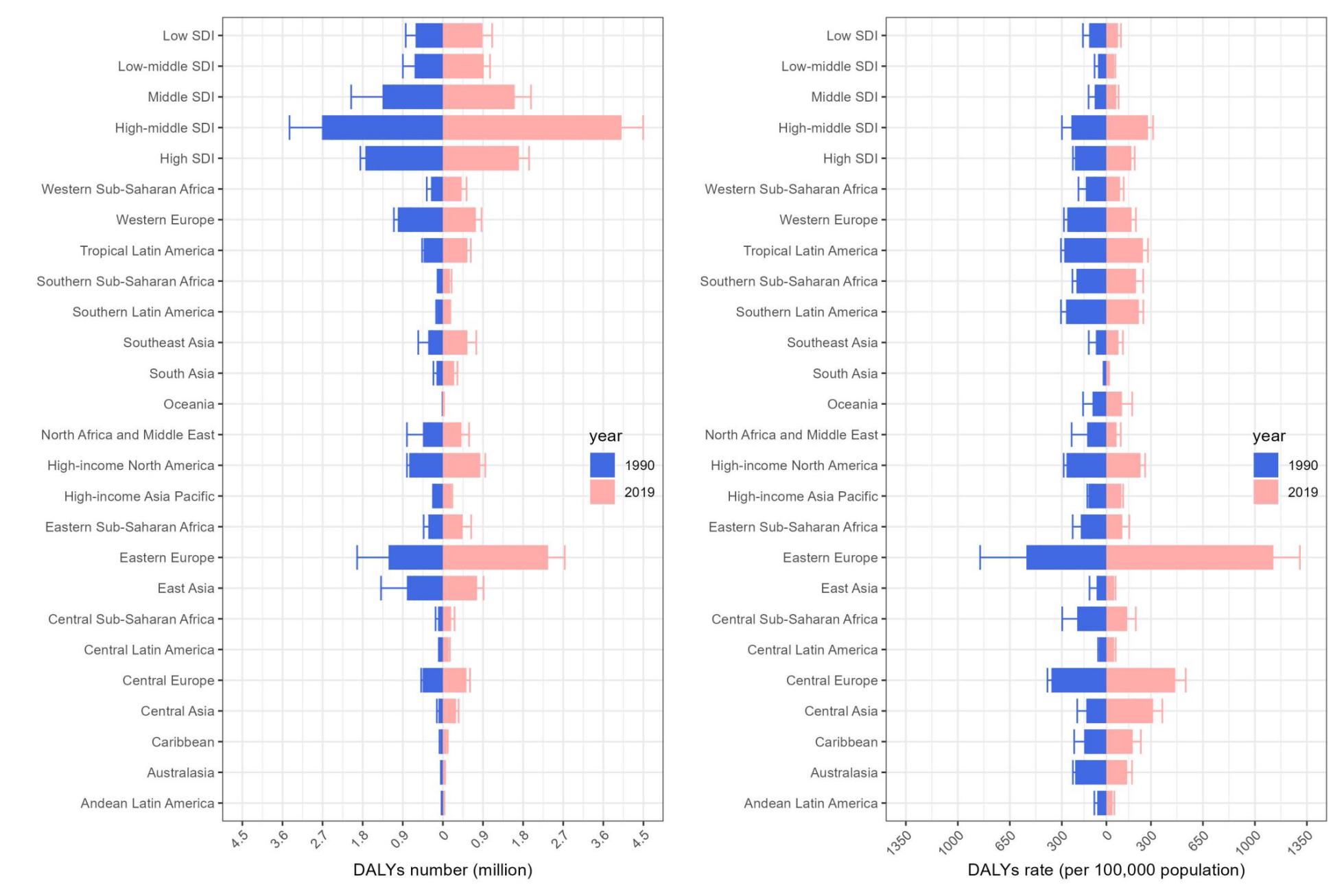
Figure S6. Change in burden of cardiomyopathy and myocarditis by region, 1990 vs 2019. Columns and error bar representing the central estimates and 95% uncertainty interval of DALYs cases (A) and DALYs rates (B) in all ages population, respectively. DALYs, disability-adjusted life-years; SDI, Sociodemographic Index.

Figure S7. Change in burden of atrial fibrillation and flutter by region, 1990 vs 2019. Columns and error bar representing the central estimates and 95% uncertainty interval of DALYs cases (A) and DALYs rates (B) in all ages population, respectively. DALYs, disability-adjusted life-years; SDI, Sociodemographic Index.


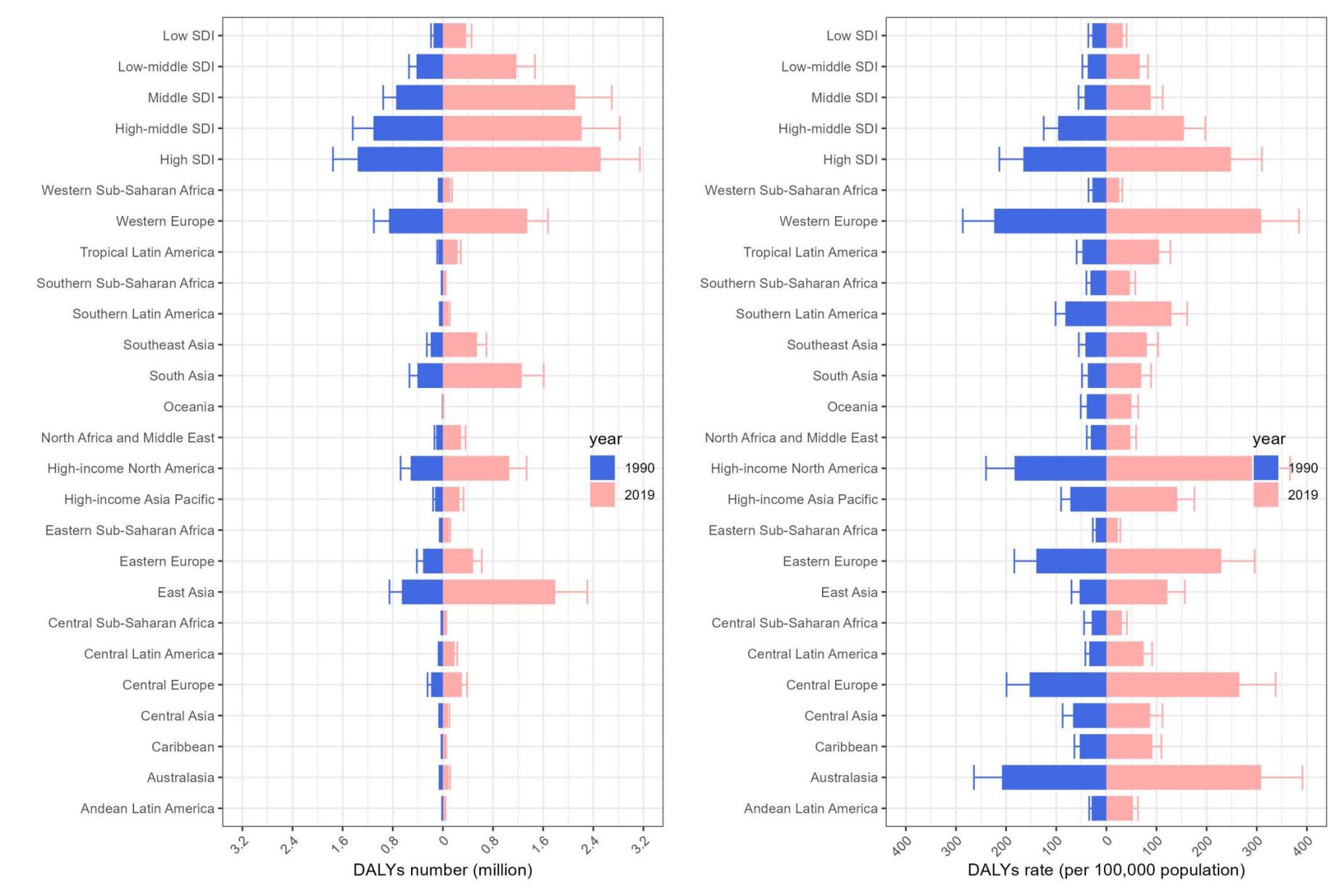


Figure S8. Change in burden of aortic aneurysm by region, 1990 vs 2019. Columns and error bar representing the central estimates and 95% uncertainty interval of DALYs cases (A) and DALYs rates (B) in all ages population, respectively. DALYs, disability-adjusted life-years; SDI, Sociodemographic Index.


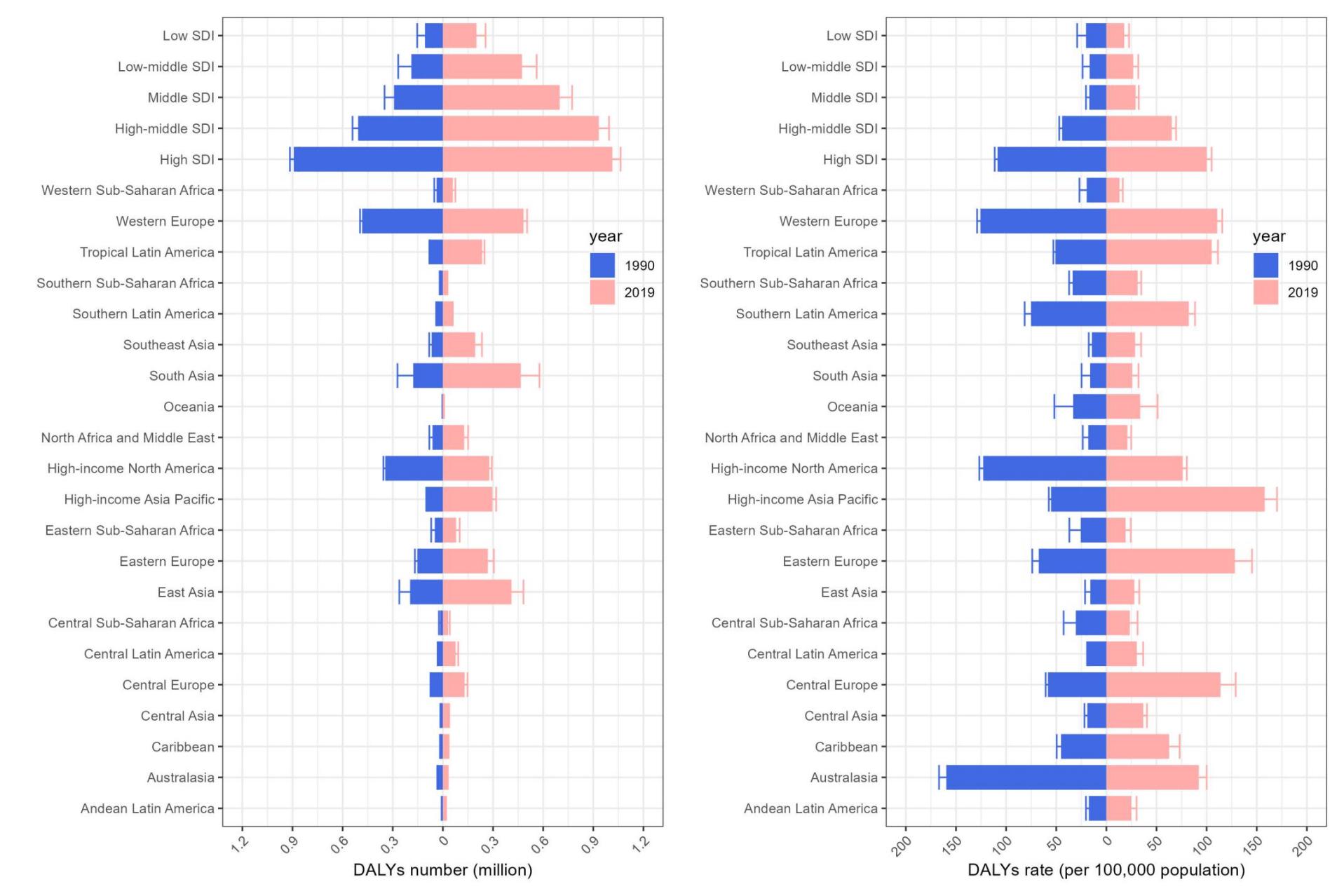


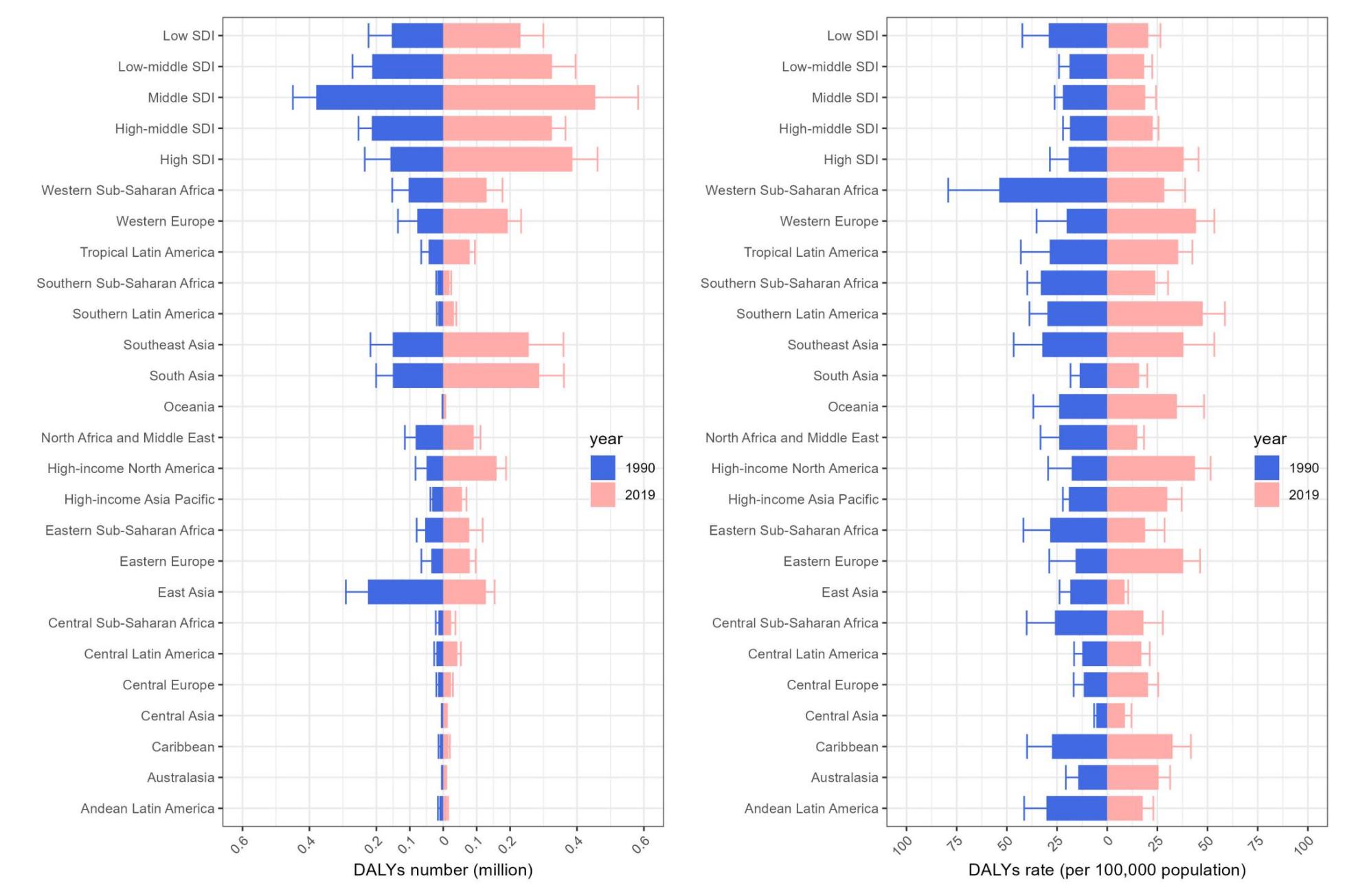
Figure S9. Change in burden of peripheral artery disease by region, 1990 vs 2019. Columns and error bar representing the central estimates and 95% uncertainty interval of DALYs cases (A) and DALYs rates (B) in all ages population, respectively. DALYs, disability-adjusted life-years; SDI, Sociodemographic Index.


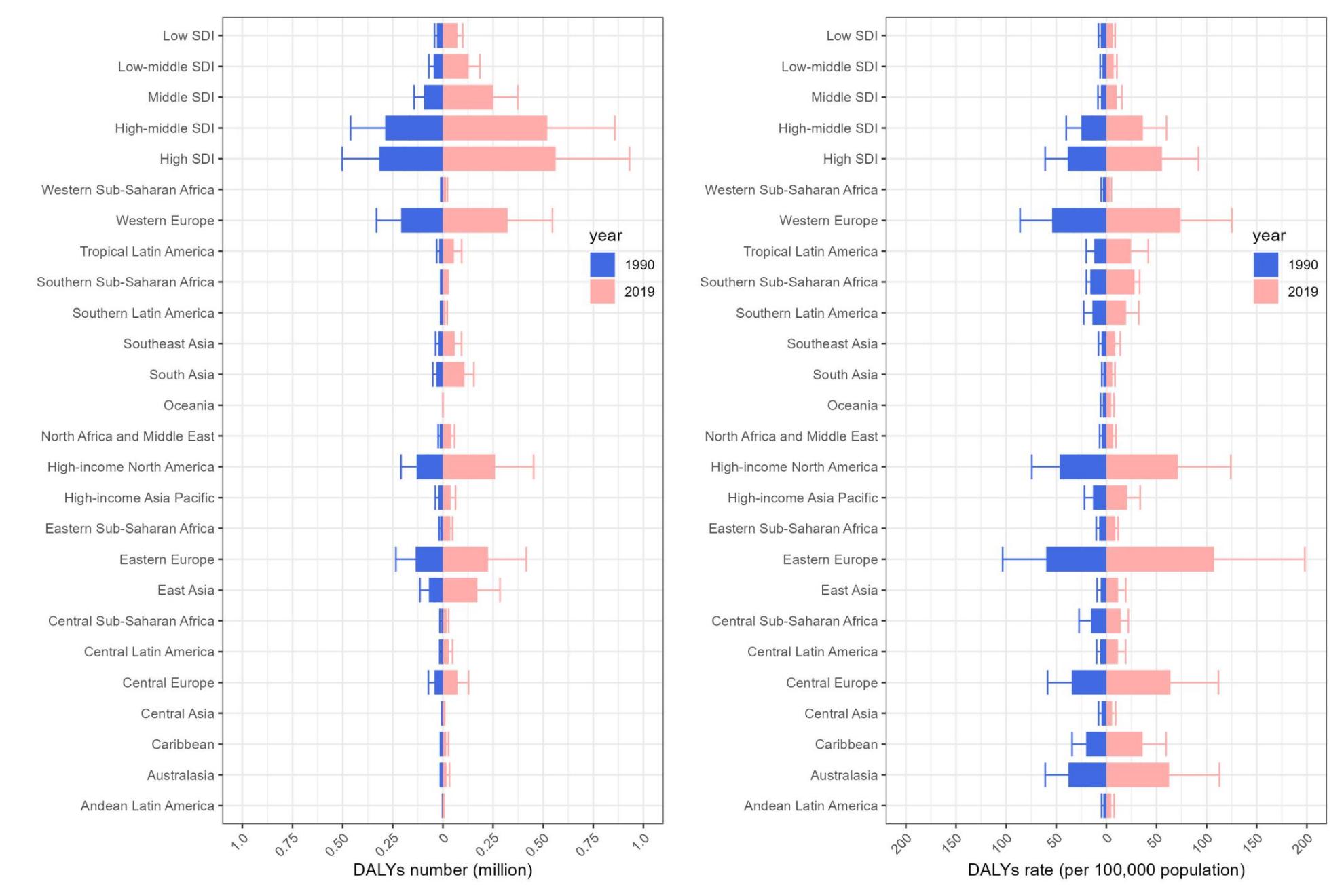
Figure S10. Change in burden of endocarditis by region, 1990 vs 2019. Columns and error bar representing the central estimates and 95% uncertainty interval of DALYs cases (A) and DALYs rates (B) in all ages population, respectively. DALYs, disability-adjusted life-years; SDI, Sociodemographic Index.
